# Surface water as a source of rare *Salmonella enterica* serovars in semiarid northeastern Brazil

**DOI:** 10.1002/jeq2.70098

**Published:** 2025-10-21

**Authors:** Alan Douglas de Lima Rocha, Daniel F. M. Monte, Laiorayne Araújo de Lima, Nádyra Jerônimo da Silva, Walter Esfrain Pereira, Patrícia Emília Naves Givisiez, Xinyang Huang, Zhao Chen, Eric W. Brown, Marc W. Allard, Rebecca L. Bell, Magaly Toro, Jianghong Meng, Celso José Bruno de Oliveira

**Affiliations:** ^1^ Departmento de Zootecnia Centro de Ciências Agrárias, Universidade Federal da Paraíba (CCA/UFPB) Areia Brazil; ^2^ Departamento de Ciências Fundamentais e Sociais, Centro de Ciências Agrárias Universidade Federal da Paraíba (UFPB) Areia Brazil; ^3^ Joint Institute for Food Safety and Applied Nutrition (JIFSAN) University of Maryland, College Park College Park Maryland USA; ^4^ Center for Food Safety and Applied Nutrition Food and Drug Administration College Park Maryland USA; ^5^ Department of Nutrition and Food Science University of Maryland College Park Maryland USA

## Abstract

*Salmonella enterica* remains a major foodborne pathogen globally but little attention has been paid to infrequent serovars in environmental settings. We report the occurrence of 30 rare *S. enterica* serovars isolated from environmental water sources between 2021 and 2022 in semiarid northeastern Brazil. We conducted two risk‐based field campaigns at shoreline access points in 10 reservoirs associated with the three largest river basins in the state. *Salmonella enterica* was recovered from 175 out of 230 water samples, yielding 2903 isolates. Of these, 938 were selected for whole‐genome sequencing (WGS). Genome assembly and downstream analyses identified 65 unique serovars, including 68 isolates belonging to 30 rare serovars. *Salmonella* Carrau (*n *= 14), *S*. Oran (*n *= 9), *S*. Gaminara (*n *= 5), and *S*. Urbana (*n *= 4) were the most frequent rare serovars. WGS analysis revealed the presence of antimicrobial resistance genes (ARGs) in all isolates. The highest abundances were associated with ARGs conferrying resistance to aminoglycosides [*aac(6′)‐Iaa* (100%)], quinolones (*parC*:p.T57S [98.1%] and *qnrB19* [3.77%]), and fosfomycin (*fosA7* [3.77%]). Some isolates carried plasmids (IncX3, IncFII [S], IncFII [Cf], Col [pHAD28], and IncFII [SARC14]) that could facilitate the spread of antimicrobial resistance. Phylogenetic analysis indicated the presence of distinct clades for each serovar. Interestingly, 20 serovars are endemic lineages circulating in Brazil, except *S*. Kiambu, which belongs to an international lineage. These findings underscore the importance of environmental monitoring and understanding the distribution of *Salmonella* in water sources to safeguard public health and prevent the spread of antimicrobial resistance.

## Core Ideas

- Rare *Salmonella enterica* serovars were recovered from surface waters in semiarid northeastern Brazil.
- Most isolates showed resistance to gentamicin and harbored multidrug efflux pump genes (mdsA/B).
- Whole‐genome sequencing revealed 22 sequence types, most reported for the first time in Brazil.
- Phylogenetic analysis showed local *Salmonella* lineages unrelated to international strains.
- Rare serovars pose overlooked risks to agrifood chains and public health in low‐income rural areas.

## Plain Language Summary

*Salmonella* is a dangerous germ that makes people sick when they eat contaminated food, but scientists do not pay much attention to rare types of this germ, which might still be harmful to people's health. We looked for *Salmonella* germs in water from lakes and rivers in northeastern Brazil, a very dry region, by collecting water samples from 10 large dams between 2021 and 2022. We discovered 30 different rare types of *Salmonella* in the water samples, with the most common rare types being Carrau, Oran, Gaminara, and Urbana, which was surprising because these types are not usually found in water. When we studied these germs more closely using advanced technology, we found that some of these *Salmonella* germs can resist medicines (antibiotics) that doctors use to treat infections, which means if someone gets sick from these germs, it might be harder to cure them. Moreover, they can transfer this resistance to other germs. Our research shows that even rare types of *Salmonella* in water sources.

**Abbreviations**
AMP: ampicillin
AXO: ceftriaxone
CFX: cefuroxime
CGE: Center for Genome Epidemiology
CHL: chloramphenicol
CIP: ciprofloxacin
eb‐PCR: enrichment broth polymerase chain reaction
ENO: enrofloxacin
ERT: ertapenem
FLF: florfenicol
GEN: gentamicin
MFX: moxifloxacin
MMS: modified Moore swabs
NAL: nalidixic acid
NCBI: National Center for Biotechnology Information
PCR: polymerase chain reaction
SNP: single nucleotide polymorphism
SXT: trimethoprim/sulfamethoxazole
TET: tetracycline
TT: tetrathionate
WGS: whole‐genome sequencing
XNL: ceftiofur

## 1 INTRODUCTION

Non‐typhoidal *Salmonella enterica* is a leading foodborne pathogen causing gastrointestinal infections in both humans and animals. While *S. enterica* has been commonly associated with contaminated food, it has been frequently reported in environmental water sources, including lakes, rivers, streams, ponds, and groundwater (Liu et al., 2018; A. D. D. L. Rocha et al., 2022). *Salmonella enterica* can enter water bodies through different pathways such as contaminated runoff, sewage and wastewater discharge, agricultural practices, and wildlife (Harris et al., 2018; B. Li et al., 2015; Toro et al., 2016), where it can survive for extended periods, especially in freshwater environments (Liu et al., 2018; Santo Domingo et al., 2000).

In the scope of a consortium led by the Joint Institute for Food Safety and Applied Nutrition, bringing together teams in the United States, Mexico, Chile, and Brazil to address *Salmonella* contamination in surface waters linked to agricultural regions in the Americas (https://jifsan.umd.edu/research/water_project), we recently demonstrated the long‐term persistence of certain *Salmonella* serovars, such as *S*. Newport and *S*. Infantis, in surface waters in different countries (Chen et al., 2024). However, water may be a source of a variety of serovars rarely or poorly described in the literature. For instance, despite *S*. Typhimurium and *S*. Enteritidis being the two most prevalent serovars related to foodborne diseases, foodstuff has been described as a source of many other rare antibiotic‐resistant *S. enterica* serovars that could not be neglected (D. F. M. Monte et al., 2021).

In Brazil, although there is an outbreak notification system (SINAN) (SINAN, 2024), there is no comprehensive assessment of salmonellosis cases. This is partly because diagnoses are often made based on clinical examination and symptomatology, followed by broad‐spectrum treatment, without conducting complementary tests to identify the etiological agent. It is also common for individuals not to seek medical assistance in milder cases. From a public health perspective, the 2022 national census indicated that only 62.5% of households are properly connected to the sewage system, with another 13.2% linked to septic tanks. This leaves 24.3% of households without adequate sewage treatment (Instituto Brasileiro De Geografia e Estatística [IBGE], 2023), inevitably increasing pollution of environmental water bodies.

Therefore, investigations on the diversity and characterization of *S. enterica* serovars contaminating natural waters are necessary for the successful assessment of the putative risks to agrifood systems and public health. This is particularly relevant for rural settings in low‐income semiarid regions, where few reservoirs that accumulate water during the rainy seasons supply water to both human populations and agricultural activities. This study aimed to discuss the public health implications of rare *S. enterica* serovars recovered from surface water reservoirs in semiarid Paraiba, northeastern Brazil, by exploring information on their prevalence, antimicrobial susceptibility patterns, and phylogenetic relatedness.

## 2 MATERIALS AND METHODS

### 2.1 Study design

**FIGURE 1:**
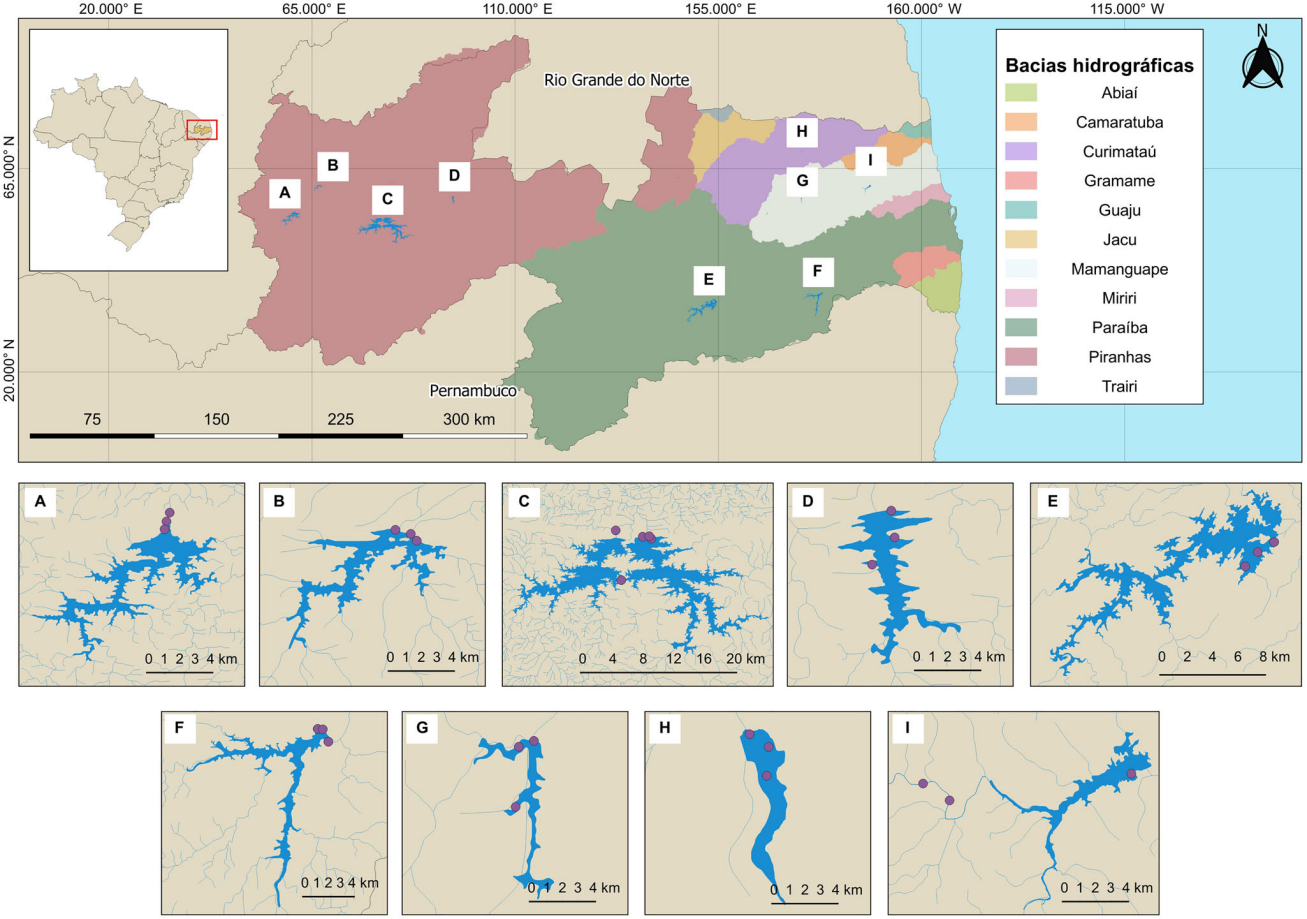
Map of the river basins in the state of Paraíba. Inserts depict the 29 sampling sites in (A) Cajazeiras, (B) Sousa, (C) Coremas and Mãe d'Água (two interconnected reservoirs), and Coremas (D) in the Piranhas river basin (violet color); in Condado (E) Boqueirão (F) Itatuba in the Paraíba river basin (green color); and (G) Areia, (H) Bananeiras, and (I) Araçagi in the Mamanguape basin river (light blue and purple colors).

We conducted two prespecified field campaigns spanning hydrologically distinct windows (February to July, 2021, and January to May, 2022) in 10 reservoirs associated with the three largest hydrographic basins in Paraíba state in semiarid region of northeastern Brazil: Piranhas, Paraíba, and Mamanguape rivers (Figure 1). To maximize the probability of detecting *S. enterica* at the human–agriculture interface, we employed a risk‐based site selection strategy: at each reservoir, three shoreline access points (sampling sites) near to irrigation withdrawal, livestock watering, or fishing were sampled monthly. At each sampling site, three successive 10‐L water samples (triplicates) were collected. A total of 230 water samples (690 swabs) were obtained. Table S1 provides the sampling dates and geographical coordinates for water sampling.

A total of 63 water samples were collected from the Mamanguape River Basin, which covers an area of 3522.69 km^2^ (Agência Executiva de Gestão de Águas [AESA], 2017, 2022). Sampling sites within this basin included two in the Araçagi Reservoir, situated along the Mamanguape River, and one site on the Jacaré River, all of them within the municipality of Araçagi. Additionally, three sampling sites were established at the Saulo Maia reservoir in the municipality of Areia, and three more at the Jandáia reservoir in the municipality of Bananeiras.

From the Paraíba River Basin, spanning 20,071.83 km^2^ (Agência Executiva de Gestão de Águas [AESA], 2017, 2022), 48 samples were collected. Despite being the second‐largest basin in the state, it plays a crucial economic role, supplying water to 52% of the state's population (Agência Executiva de Gestão de Águas [AESA], 2017). Sampling sites included three sites in the Epitácio Pessoa reservoir, the state's second‐largest reservoir with a capacity of 466 million m^3^. Two additional sampling sites were located in the Acauã reservoir, the fourth largest in the state, with a capacity of 253 million m^3^. One additional sampling site was established along the Paraíba River itself.

The Piranhas River Basin, the largest in the state at 26,047.49 km^2^ (Agência Executiva de Gestão de Águas [AESA], 2017, 2022), contributed 119 samples. In the municipality of Coremas, samples were taken from three sites in the Coremas reservoir and two in the Mãe D’água reservoir. These two interconnected reservoirs form the largest water storage complex in the state, with a combined capacity of 1.289 billion m^3^. Three additional sites were sampled from the São Gonçalo reservoir in the municipality of Sousa. Further three sites are located in the Engenheiro Ávidos reservoir in Cajazeiras, the third largest in the state with a capacity of 293 million m^3^. Finally, three sampling sites were established in the Engenheiro Arcoverde reservoir in the municipality of Condado.

**Core Ideas**

- Rare *Salmonella enterica* serovars were recovered from surface waters in semiarid northeastern Brazil.
- Most isolates showed resistance to gentamicin and harbored multidrug efflux pump genes (mdsA/B).
- Whole‐genome sequencing revealed 22 sequence types, most reported for the first time in Brazil.
- Phylogenetic analysis showed local *Salmonella* lineages unrelated to international strains.
- Rare serovars pose overlooked risks to agrifood chains and public health in low‐income rural areas.

### 2.2 Water sampling

The water sample procedure involved the use of modified Moore swabs (MMS) prepared as previously described (Sbodio et al., 2013). Briefly, a 0.9 m2 folded cheesecloth grade #90 was tightly rolled into an assembled apparatus (MMS‐cassette) consisting of a 10‐cm‐long polyvinyl chloride tube with a male‐to‐male coupler and a connector at each end. The assembly resulted in a filtration cassette unit (FCU), providing a cylindrical‐shaped swab as a filtering matrix. FCUs were individually packed and sterilized in an autoclave. At the sampling points, the cassettes were unpacked and attached to a portable peristaltic pump (CPD‐201‐3, MS TECNOPON Equipamentos Especiais LTDA) using a sterile latex tube. A volume of 10 L of water was filtered for a period of 20 min at a rate of 500 mL per minute. The filtration matrices were transferred aseptically into sterile containers with 250 mL of modified buffered peptone water (1.25 g of sodium chloride, 0.875 g of disodium hydrogen phosphate, and 0.375 g of potassium dihydrogen phosphate). The containers were refrigerated at 4°C and transported to the laboratory. The triplicates from each sampling site were acquired by doing three successive samplings at each site utilizing individual sterile cassettes. The sampling sites' geographic coordinates were documented using the Epicollect application on an Android device (Aanensen et al., 2014).

### 2.3 Microbiological procedures

*Salmonella* isolation was performed according to the *Bacteriological Analytical Manual* protocol (Andrews et al., 2024) with modifications. Briefly, pre‐enrichment was performed at 37 ± 1°C for 24 h. Aliquots (0.1 mL) were transferred into 9.9 mL Rappaport–Vassiliadis (RV) broth (Oxoid, Thermo Fisher Scientific), while 1‐mL aliquots were transferred into 9 mL tetrathionate (TT) broth (Oxoid, Thermo Fisher Scientific). Broths were enriched at 42.5 ± 1°C for 18 h in a water bath. A loopful of TT‐ and RV‐enriched broths was streaked onto XLT‐4 Agar (Oxoid, Thermo Fisher Scientific). The MMS workflow employed a selective enrichment step because expected *Salmonella* levels in surface water were very low; therefore, results were qualitative (presence/absence) rather than quantitative.

In parallel, we performed an enrichment broth polymerase chain reaction (eb‐PCR) targeting the *Inv*A gene for detecting *Salmonella*‐positive samples. DNA was extracted by the boiling‐centrifugation method as previously reported (Freschi et al., 2005). Enriched broth (1.5 mL) was centrifuged at 12,000 g for 2 min. The resulting supernatant was discarded, and the pellet was resuspended in 800 µL of ultrapure water, followed by a further centrifugation step at 12,000 g for 2 min. Subsequently, the supernatant was removed, and the pellet was resuspended in 200 µL of ultrapure water and heated at 95°C for 10 min. A final centrifugation step (12,000 rpm for 2 min) was performed, and 100 µL supernatant was transferred into a DNase‐free sterile microtube.

Polymerase chain reaction (PCR) assay used the species‐specific primers (Forward: 5′ GTG AAA TTA TCG CCA CGT TCG GGC AA 3′ and Reverse: 5′ TCA TCG CAC CGT CAA AGG AAC C 3′) targeting the *Inv*A gene in *Salmonella* spp. PCR was performed in a 25 µL master mix final volume containing 1.4 mM MgCl2, 1.25 U DNA Polymerase (Platus Taq, Sinapse Inc.), 2 mM of each dNTP, 10 µM of each primer, 1 µg DNA, and 10X Taq Buffer (KCl). The DNA amplification was performed in a thermal cycler (Biometra T‐Advanced, Analytik Jena AG) with the following cycling conditions: an initial denaturation (95°C; 3 min), followed by 35 cycles consisting of a denaturation step (95°C for 30 s), annealing (53°C for 30 s), and extension (72°C for 2 min and 20 s), and a final extension step (72°C for 5 min). The resulting PCR products were electrophorized on a 1.5% agarose gel at 80 V for 40 min in 1x tris‐acetate EDTA buffer, stained with SYBR Safe DNA Gel Stain, and visualized under ultraviolet light (Gel Logic 212 PRO, Carestream Health).

Positive broths in the be‐PCR assay were further streaked onto Hektoen Enteric Agar (Oxoid) and Bismuth Sulfite Agar (Oxoid). The agar plates were incubated at 37 ± 1°C for 24 h. Up to three typical *Salmonella* colonies from each plate were selected, giving priority to morphological diversity. Selected colonies were inoculated on triple sugar iron (Oxoid) agar and lysine iron agar (Oxoid) slants for biochemical tests, which were further incubated at 37 ± 1°C for 16 h. Presumptive *Salmonella* isolates were streaked onto Tryptic Soy Agar (Oxoid) followed by PCR confirmation, which was performed using a loopful of bacterial mass mixed in 100 µL of DNase/RNase‐free water as a DNA template.

PCR‐confirmed isolates were incubated in brain heart infusion broth (Oxoid) supplemented with 20% glycerol (Neon Comercial Reagentes Analíticos LTDA) and stored at −70°C.

### 2.4 *Salmonella* strains and serotyping

For the selection criteria, we defined as rare or infrequent the serovars not listed in “An Atlas of *Salmonella* in the United States, 1968–2011” (CDC, 2013), as well as those not ranked among the most frequent serovars associated with human salmonellosis in Brazil from 2011 to 2020 (Santos et al., 2022) (Table 1).

**TABLE 1:** List of all identified *Salmonella enterica* serovars recovered from surface water samples in 204 Paraíba, northeastern Brazil.

Serovars reported in the CDC Atlas^a^ or Santos et al. (2022)^b^	Serovars classified as rare
Saintpaul	Oran
Newport	Carrau
Infantis	Urbana
Javiana	Gaminara
Poona	Freetown
Braenderup	Businga
Sandiego	Molade or Wippra
Muenchen	Bullbay
Agona	Kiambu
Hadar	Lomita
Anatum	Adelaide
Schwarzengrund	Othmarschen
Mbandaka	Langenhorn
Panama	Lille
Thompson	Mikawasima
Oranienburg	Somone or IV 6,7:z4,z24:‐
Rubislaw	I 16:e,h:e,n,z15
Corvallis	I 18:d:‐
Albany or Duesseldorf	I 7:l,v:‐
Potsdam	I 4:b:‐
Muenster	I 4:‐:1,5
Cerro	I 7:k:‐
Michigan	I 7:‐:1,5
Brandenburg	I 16:r:e,n,z15
Farmsen or Poona	IV [1],53:g,z51:‐
Meleagridis	IV 50:z4,z23:‐
Minnesota	I 3,10:d:‐
Ohio	IV 45:g,z51:‐
Pomona	I ‐:l,v:e,n,z15
Coeln	II 43:z4,z23:‐ or IIIa 43:z4,z23:‐ or Farmingdale or IV 43:z4,z23:‐
Glostrup or Chomedey	
Inganda	
I 1,3,19:b:‐	
II 42:r:‐	
I 1,3,19:c:‐	

*Note*: Serovars in the left column have been reported in the CDC 205 Atlas or by Santos et al. (2022) as frequently reported serovars associated with foodborne 206 illness in the United States and Brazil. Serovars in the right column were classified as rare; they do not appear among serovars reported in the CDC Atlas or the survey by Santos et al. (2022).

^a^ CDC, 2013.

^b^ Santos et al. (2022).

### 2.5 Antimicrobial susceptibility testing

All *S. enterica* strains were tested for antimicrobial susceptibility testing against critically important antimicrobials, including amoxicillin/clavulanic acid 2:1 ratio, ceftiofur (XNL), ceftriaxone (AXO), cefuroxime (CFX), ampicillin (AMP), trimethoprim/sulfamethoxazole (SXT), chloramphenicol (CHL), florfenicol (FLF), ciprofloxacin (CIP), ofloxacin, nalidixic acid (NAL), moxifloxacin (MFX), enrofloxacin (ENO), tetracycline (TET), gentamicin (GEN), and ertapenem (ERT). Zone diameters were interpreted according to the guidelines of the Clinical and Laboratory Standards Institute (CLSI, 2023b) and the VET01S (CLSI, 2023a). Multidrug resistance was defined as resistant to three or more classes of antimicrobials.

### 2.6 Genomic sequencing and data analysis

*S. enterica* isolates were whole‐genome sequenced at the Center for Food Safety and Applied Nutrition, FDA. After DNA extraction using a commercial kit (DNA Blood and Tissue kit, Qiagen), the DNA libraries were prepared with the Illumina DNA prep kit (Illumina) and paired‐end sequenced (2 × 150 bp) using a NextSeq 2000 platform (Illumina). Reads were submitted as FASTQ files to the Sequence Read Archive, National Center for Biotechnology Information (NCBI), Bioprojects PRJNA186035 and PRJNA560080 (Table 2). Fastq files were uploaded into CLC Genomics Workbench (CLC Bio, Qiagen) to check the quality of the sequences and ensure the non‐contamination of the reads. Afterwards, de novo assembly was performed using the same software. All sequences have been deposited at the NCBI, and their accession numbers are listed in Table 1.

**TABLE 2:** Genomic features of rarely isolated *Salmonella enterica* serovars recovered from surface waters in Paraiba state, northeastern Brazil.

Accession number	Serovar by WGS	Source	Resistance genes	Sequence type	R‐type	Location	Plasmid
GCA_023495885.1	Carrau	Reservoir water	*mdsA*, *mdsB*, *aac*(*6′*)*‐Iaa*, *parC:p.T57S*	226	Pan‐susceptible	7°25′56.5″ S 35°33′41.7″ W	IncX3
GCA_023733395.1	Carrau	Reservoir water	*mdsA*, *mdsB*, *aac*(*6′*)*‐Iaa*, *parC:p.T57S*	226	Pan‐susceptible	6°51′16.4″ S 35°18′03.9″ W	None
GCA_025402575.1	Carrau	Reservoir water	*mdsA*, *mdsB*, *aac*(*6′*)*‐Iaa*, *parC:p.T57S*	226	GEN	6°39′33.8″ S 35°40′34.9″ W	None
GCA_025401895.1	Carrau	Reservoir water	*mdsA*, *mdsB*, *aac*(*6′*)*‐Iaa*, *parC:p.T57S*	226	GEN	6°39′30.6″ S 35°40′39.7″ W	None
GCA_025512195.1	Carrau	Reservoir water	*mdsA*, *mdsB*, *aac*(*6′*)*‐Iaa*, *parC:p.T57S*	226	Pan‐susceptible	6°59′13.3″ S 38°27′19.0″ W	IncFII (S)
GCA_029753455.1	Carrau	River	*mdsA*, *mdsB*, *aac*(*6′*)*‐Iaa*, *parC:p.T57S*	226	Pan‐susceptible	7°26′38.2″ S 35°33′36.7″ W	None
GCA_029673985.1	Carrau	Reservoir water	*mdsA*, *mdsB*, *aac*(*6′*)*‐Iaa*, *parC:p.T57S*	226	Pan‐susceptible	6°54′53.3″ S 37°35′04.1″ W	None
GCA_029673945.1	Carrau	Reservoir water	*mdsA*, *mdsB*, *aac*(*6′*)*‐Iaa*, *parC:p.T57S*	226	Pan‐susceptible	6°59′29.6″ S 38°27′23.0″ W	IncFII (S)
GCA_029667365.1	Carrau	Reservoir water	*mdsA*, *mdsB*, *aac*(*6′*)*‐Iaa*, *parC:p.T57S*	226	GEN	6°55′17.8″ S 37°35′25.2″ W	None
GCA_029668105.1	Carrau	River	*mdsA*, *mdsB*, *aac*(*6′*)*‐Iaa*, *parC:p.T57S*	226	GEN	6°51′47.1″ S 35°21′32.1″ W	None
GCA_029744325.1	Carrau	Reservoir water	*mdsA*, *mdsB*, *aac*(*6′*)*‐Iaa*, *parC:p.T57S*	226	GEN	7°25′56.5″ S 35°33′41.7″ W	IncX3
GCA_029746395.1	Carrau	Reservoir water	*mdsA*, *mdsB*, *aac*(*6′*)*‐Iaa*, *parC:p.T57S*	226	GEN	7°01′26.4″ S 37°57′11.1″ W	None
GCA_029748545.1	Carrau	River	*mdsA*, *mdsB*, *aac*(*6′*)*‐Iaa*, *parC:p.T57S*	226	GEN	6°51′27.8″ S 35°22′02.5″ W	None
GCA_029751595.1	Carrau	Reservoir water	*mdsA*, *mdsB*, *aac*(*6′*)*‐Iaa*, *parC:p.T57S*	226	Pan‐susceptible	6°51′16.4″ S 35°18′03.9″ W	None
GCA_029755455.1	Oran	Reservoir water	*mdsA*, *mdsB*, *aac*(*6′*)*‐Iaa*, *parC:p.T57S*	965	Pan‐susceptible	6°59′29.6″ S 38°27′23.0″ W	None
GCA_029754375.1	Oran	Reservoir water	*mdsA*, *mdsB*, *aac*(*6′*)*‐Iaa*, *parC:p.T57S*	965	GEN	6°50′45.1″ S 38°18′40.1″ W	None
GCA_029756695.1	Oran	Reservoir water	*mdsA*, *mdsB*, *aac*(*6′*)*‐Iaa*, *parC:p.T57S*	965	Pan‐susceptible	6°59′13.3″ S 38°27′19.0″ W	None
GCA_029754235.1	Oran	Reservoir water	*mdsA*, *mdsB*, *aac*(*6′*)*‐Iaa*, *parC:p.T57S*	965	GEN	6°59′13.3″ S 38°27′19.0″ W	None
GCA_029755175.1	Oran	River	*mdsA*, *mdsB*, *aac*(*6′*)*‐Iaa*, *parC:p.T57S*	965	GEN	6°58′57.6″ S 38°27′12.1″ W	None
GCA_029746255.1	Oran	Reservoir water	*mdsA*, *mdsB*, *aac*(*6′*)*‐Iaa*, *parC:p.T57S*	965	GEN	6°59′29.6″ S 38°27′23.0″ W	None
GCA_029745175.1	Oran	Reservoir water	*mdsA*, *mdsB*, *aac*(*6′*)*‐Iaa*, *parC:p.T57S*	965	GEN	6°59′29.6″ S 38°27′23.0″ W	None
GCA_029745155.1	Oran	River	*mdsA*, *mdsB*, *aac*(*6′*)*‐Iaa*, *parC:p.T57S*	965	GEN	6°58′57.6″ S 38°27′12.1″ W	None
GCA_029744645.1	Oran	River	*mdsA*, *mdsB*, *aac*(*6′*)*‐Iaa*, *parC:p.T57S*	965	GEN	6°58′57.6″ S 38°27′12.1″ W	None
GCA_023800495.1	Urbana	Reservoir water	*mdsA*, *mdsB*, *aac*(*6′*)*‐Iaa*, *parC:p.T57S*	754	GEN	6°51′22.5″ S 38°21′05.5″ W	IncFII (Cf)
GCA_025513125.1	Urbana	Reservoir water	*mdsA*, *mdsB*, *aac*(*6′*)*‐Iaa*, *parC:p.T57S*	754	Pan‐susceptible	6°59′13.3″ S 38°27′19.0″ W	IncX3
GCA_023495205.1	Urbana	Reservoir water	*mdsA*, *mdsB*, *aac*(*6′*)*‐Iaa*, *parC:p.T57S*	754	GEN	6°51′22.5″ S 38°21′05.5″ W	IncFII (Cf)
GCA_029667725.1	Urbana	Reservoir water	*mdsA*, *mdsB*, *aac*(*6′*)*‐Iaa*, *parC:p.T57S*	754	Pan‐susceptible	7°01′34.9″ S 37°56′36.7″ W	IncX3
GCA_029673145.1	Gaminara	Reservoir water	*mdsA*, *mdsB*, *aac*(*6′*)*‐Iaa*, *parC:p.T57S*	239	GEN	6°39′35.5″ S 35°40′34.0″ W	None
GCA_029672805.1	Gaminara	Reservoir water	*mdsA*, *mdsB*, *aac*(*6′*)*‐Iaa*, *parC:p.T57S*	9398	GEN	7°25′57.0″ S 35°33′39.2″ W	IncFII (S)
GCA_029747085.1	Gaminara	Reservoir water	*mdsA*, *mdsB*, *aac*(*6′*)*‐Iaa*, *parC:p.T57S*	9398	GEN	7°25′57.0″ S 35°33′39.2″ W	IncFII (S)
GCA_023495185.1	Gaminara	River	*mdsA*, *mdsB*, *aac*(*6′*)*‐Iaa*, *parC:p.T57S*	239	GEN	7°26′38.2″ S 35°33′36.7″ W	None
GCA_023494005.1	Gaminara	Reservoir water	*mdsA*, *mdsB*, *aac*(*6′*)*‐Iaa*, *parC:p.T57S*	9398	GEN	7°25′57.0″ S 35°33′39.2″ W	IncFII (S)
GCA_025512175.1	Lille	Reservoir water	*mdsA*, *mdsB*, *aac*(*6′*)*‐Iaa*, *parC:p.T57S*	9792	Pan‐susceptible	6°54′28.3″ S 37°35′07.3″ W	None
GCA_025511735.1	Freetown	Reservoir water	*mdsA*, *mdsB*, *aac*(*6′*)*‐Iaa*, *parC:p.T57S*	2940	Pan‐susceptible	7°01′27.4″ S 37°57′11.8″ W	IncFII (S)
GCA_025511215.1	Businga	Reservoir water	*mdsA*, *mdsB*, *aac*(*6′*)*‐Iaa*, *parC:p.T57S*	9793	GEN	6°50′45.1″ S 38°18′40.1″ W	None
GCA_025550195.1	Bullbay	Reservoir water	*mdsA*, *mdsB*, *aac*(*6′*)*‐Iaa*, *parC:p.T57S*	9402	GEN	6°50′45.1″ S 38°18′40.1″ W	None
GCA_029674085.1	Molade or Wippra	River	*qnrB19*, *parC:p.T57S*, *fosA7*, *mdsA*, *mdsB*	544	NAL‐GEN	7°01′00.3″ S 37°59′07.8″ W	Col (pHAD28)
GCA_029668905.1	Kiambu	River	*qnrB19*, *aac*(*6′*)*‐Iaa*, *mdsA*, *mdsB*	309	NAL‐GEN‐ENO	7°01′00.3″ S 37°59′07.8″ W	Col (pHAD28)
GCA_029745735.1	Lomita	River	*mdsA*, *mdsB*, *aac*(*6′*)*‐Iaa*, *parC:p.T57S*	Unknown	GEN	6°51′27.8″ S 35°22′02.5″ W	None
GCA_029745815.1	Mikawasima	River	*mdsA*, *mdsB*, *aac*(*6′*)*‐Iaa*, *parC:p.T57S*	Unknown	Pan‐susceptible	6°51′27.8″ S 35°22′02.5″ W	None
GCA_029671665.1	Langenhorn	River	*aac*(*6′*)*‐Iaa*, *parC:p.T57S*	Unknown	Pan‐susceptible	7°26′38.2″ S 35°33′36.7″ W	IncFII (SARC14)
GCA_023802655.1	Othmarschen	River	*mdsA*, *mdsB*, *aac*(*6′*)*‐Iaa*, *parC:p.T57S*	9457	GEN	6°58′57.6″ S 38°27′12.1″ W	None
GCA_029753435.1	Adelaide	Reservoir water	*mdsA*, *mdsB*, *aac*(*6′*)*‐Iaa*, *parC:p.T57S*	440	GEN	6°50′51.6″ S 38°18′33.7″ W	None
GCA_029673665.1	I 16:e,h:e,n,z15	Reservoir water	*mdsA*, *mdsB*, *aac*(*6′*)*‐Iaa*, *parC:p.T57S*	Unknown	GEN	6°39′30.3″ S 35°40′39.4″ W	None
GCA_029667845.1	I 16:e,h:e,n,z15	Reservoir water	*mdsA*, *mdsB*, *aac*(*6′*)*‐Iaa*, *parC:p.T57S*	Unknown	Pan‐susceptible	6°39′30.3″ S 35°40′39.4″ W	None
GCA_029673125.1	I 16:e,h:e,n,z15	Reservoir water	*mdsA*, *mdsB*, *aac*(*6′*)*‐Iaa*, *parC:p.T57S*	Unknown	Pan‐susceptible	6°39′30.3″ S 35°40′39.4″ W	None
GCA_029673065.1	I 16:e,h:e,n,z15	Reservoir water	*mdsA*, *mdsB*, *aac*(*6′*)*‐Iaa*, *parC:p.T57S*	Unknown	GEN	6°39′30.3″ S 35°40′39.4″ W	None
GCA_029746135.1	II 43:z4,z23:‐ or IIIa 43:z4,z23:‐ or Farmingdale or IV 43:z4,z23:‐	Reservoir water	*aac*(*6′*)*‐Iaa*, *parC:p.T57S*	762	Pan‐susceptible	6°51′22.5″ S 38°21′05.5″ W	None
GCA_029752115.1	II 43:z4,z23:‐ or IIIa 43:z4,z23:‐ or Farmingdale or IV 43:z4,z23:‐	River	*aac*(*6′*)*‐Iaa*, *parC:p.T57S*	3942	GEN	6°51′27.8″ S 35°22′02.5″ W	None
GCA_029755295.1	II 43:z4,z23:‐ or IIIa 43:z4,z23:‐ or Farmingdale or IV 43:z4,z23:‐	Reservoir water	*aac*(*6′*)*‐Iaa*, *parC:p.T57S*	3942	GEN	6°51′16.4″ S 35°18′03.9″ W	None
GCA_029668585.1	I 18:d:‐	Reservoir water	*mdsA*, *mdsB*, *aac*(*6′*)*‐Iaa*, *parC:p.T57S*	Unknown	GEN	6°54′28.3″ S 37°35′07.3″ W	None
GCA_029663955.1	I 18:d:‐	Reservoir water	*mdsA*, *mdsB*, *aac*(*6′*)*‐Iaa*, *parC:p.T57S*	Unknown	GEN	6°55′17.8″ S 37°35′25.2″ W	None
GCA_029667445.1	I 18:d:‐	Reservoir water	*mdsA*, *mdsB*, *aac*(*6′*)*‐Iaa*, *parC:p.T57S*	Unknown	Pan‐susceptible	6°54′28.3″ S 37°35′07.3″ W	None
GCA_023496145.1	I 7:l,v:‐	River	*mdsA*, *mdsB*, *aac*(*6′*)*‐Iaa*, *parC:p.T57S*	9397	GEN	6°51′27.8″ S 35°22′02.5″ W	None
GCA_025402055.1	I 7:l,v:‐	Reservoir water	*mdsA*, *mdsB*, *aac*(*6′*)*‐Iaa*, *parC:p.T57S*	Unknown	GEN	6°39′33.8″ S 35°40′34.9″ W	None
GCA_025537495.1	I 7:l,v:‐	River	*mdsA*, *mdsB*, *aac*(*6′*)*‐Iaa*, *parC:p.T57S*	9397	GEN	6°51′27.8″ S 35°22′02.5″ W	None
GCA_029668525.1	I 4:b:‐	River	*mdsA*, *mdsB*, *aac*(*6′*)*‐Iaa*, *parC:p.T57S*	Unknown	GEN	7°01′00.3″ S 37°59′07.8″ W	None
GCA_029663845.1	I 4:b:‐	River	*mdsA*, *mdsB*, *aac*(*6′*)*‐Iaa*, *parC:p.T57S*	Unknown	Pan‐susceptible	7°01′00.3″ S 37°59′07.8″ W	None
GCA_023729115.1	I 4:‐:1,5	Reservoir water	*aac*(*6′*)*‐Iaa*, *parC:p.T57S*, *fosA7*, *mdsA*, *mdsB*	9456	Pan‐susceptible	6°54′28.3″ S 37°35′07.3″ W	None
GCA_029663575.1	I 7:k:‐	Reservoir water	*mdsA*, *mdsB*, *aac*(*6′*)*‐Iaa*, *parC:p.T57S*	Unknown	GEN	6°55′17.8″ S 37°35′25.2″ W	None
GCA_029744205.1	I 7:‐:1,5	Reservoir water	*mdsA*, *mdsB*, *aac*(*6′*)*‐Iaa*, *parC:p.T57S*	Unknown	GEN	7°25′57.0″ S 35°33′39.2″ W	None
GCA_025401955.1	I 16:r:e,n,z15	Reservoir water	*mdsA*, *mdsB*, *aac*(*6′*)*‐Iaa*, *parC:p.T57S*	Unknown	GEN	6°39′30.6″ S 35°40′39.7″ W	None
GCA_025540175.1	l,v:e,n,z15	Reservoir water	*mdsA*, *mdsB*, *aac*(*6′*)*‐Iaa*, *parC:p.T57S*	9402	GEN	7°25′56.5″ S 35°33′41.7″ W	None
GCA_023496625.1	IV [1],53:g,z51:‐	River	*aac*(*6′*)*‐Iaa*, *parC:p.T57S*	9399	Pan‐susceptible	7°26′38.2″ S 35°33′36.7″ W	None
GCA_029670665.1	IV 50:z4,z23:‐	Reservoir water	*aac*(*6′*)*‐Iaa*, *parC:p.T57S*	433	Pan‐susceptible	6°39′30.3″ S 35°40′39.4″ W	None
GCA_029744485.1	I 3,10:d:‐	River	*mdsA*, *mdsB*, *aac*(*6′*)*‐Iaa*, *parC:p.T57S*	2742	GEN	6°51′27.8″ S 35°22′02.5″ W	None
GCA_029751555.1	Somone or IV 6,7:z4,z24:‐	River	*aac*(*6′*)*‐Iaa*, *parC:p.T57S*	162	GEN	7°26′38.2″ S 35°33′36.7″ W	None
GCA_029755335.1	IV 45:g,z51:‐	River	*aac*(*6′*)*‐Iaa*, *parC:p.T57S*	Unknown	Pan‐susceptible	7°26′38.2″ S 35°33′36.7″ W	None

Abbreviations: ENO, enrofloxacin; GEN, gentamicin; NAL, nalidixic acid; WGS, whole‐genome sequencing.

All isolates were serotyped in silico using default settings in SeqSero 1.2 (http://www.genomicepidemiology.org/).

Additionally, we used AMRFinderPlus version 3.11.11 with database version 2023‐04‐17.1 to identify antimicrobial resistance genes and point mutations in the assemblies (Feldgarden et al., 2021). Plasmidome, and multi‐locus sequence typing profiles were determined using PlasmidFinder 2.1, and MLST 2.0 databases (where MLST is multi‐locus sequence typing), respectively, which are available at the Center for Genome Epidemiology (CGE) platform (http://www.genomicepidemiology.org/).

### 2.7 Phylogenetic analysis

A single nucleotide polymorphism (SNP)‐based phylogeny was reconstructed from the rare *S*. *enterica* isolates (*n* = 68) using CSI Phylogeny v1.4 (Kaas et al., 2014) in the CGE (https://cge.cbs.dtu.dk/services/CSIPhylogeny/) with default settings. Briefly, assemblies were mapped and high‐quality SNPs were called and filtered using the pipeline's defaults (minimum depth ≥10, SNP quality ≥30, mapping quality ≥25, minimum inter‐SNP distance ≥10 bp, and ambiguous/indel positions removed). The resulting high‐confidence SNPs were concatenated into a core alignment sites, of which were variable. For display and support values, we re‐inferred a maximum‐likelihood tree from this SNP alignment using IQ‐TREE 2 with ModelFinder (best‐fit GTR+G), 1000 ultrafast bootstrap replicates, and 1000 Shimodaira‐Hasegawa approximate Likelihood Ratio Test (SH‐aLRT) replicates; node labels show SH‐aLRT/UFBoot where space allows. The final tree was midpoint‐rooted and annotated in iTOL v6.

### 2.8 Statistical analysis

We used descriptive statistics to report *S. enterica* detection frequencies by campaign, source (reservoir), and sampling site.

## 3 RESULTS

### 3.1 *Salmonella enterica* serovars

*S. enterica* was recovered from 175 out of 230 water samples (76.1%), yielding 2903 isolates. Of these, 938 were selected for whole genome sequencing. Genome assembly and downstream analyses identified 65 different serovars (Table S1). Among these, 68 isolates (7.24%) belonged to 30 infrequent serovars (Table 1): *S*. Carrau (*n *= 14), *S*. Oran (*n *= 9), *S*. Gaminara (*n *= 5), *S*. Urbana (*n *= 4), *S*. I 16:e,h:e,n,z15 (*n *= 4), *S*. II 43:z4,z23:‐ or IIIa 43:z4,z23:‐ or Farmingdale or IV 43:z4,z23:‐ (*n *= 3), *S*. I 18:d:‐ (*n *= 3), *S*. I 7:l,v:‐ (*n *= 3), *S*. I 4:b:‐ (*n *= 2), *S*. Lille (*n *= 1), *S*. Freetown (*n *= 1), *S*. Businga (*n *= 1), *S*. Bullbay (*n *= 1), *S*. Molade or Wippra (*n *= 1), *S*. Kiambu (*n *= 1), *S*. Lomita (*n *= 1), *S*. Mikawasima (*n *= 1), *S*. Langenhorn (*n *= 1), *S*. Othmarschen (*n *= 1), *S*. Adelaide (*n *= 1), *S*. I 4:‐:1,5 (*n *= 1), *S*. I 7:k:‐ (*n *= 1), *S*. I 7:‐:1,5 (*n *= 1), *S*. I 16:r:e,n,z15 (*n *= 1), *S*. l,v:e,n,z15 (*n *= 1), *S*. IV [1],53:g,z51:‐ (*n *= 1), *S*. IV 50:z4,z23:‐ (*n *= 1), *S*. I 3,10:d:‐ (*n *= 1), *S*. Somone or IV 6,7:z4,z24:‐ (*n *= 1), and *S*. IV 45:g,z51:‐ (*n *= 1). Among the 68 isolates obtained from 2021 to 2022, 46 originated from reservoirs and 22 from rivers.

### 3.2 Antimicrobial susceptibility testing

All strains were susceptible to amoxicillin/clavulanic acid, XNL, AXO, CFX, AMP, SXT, CHL, FLF, CIP, MFX, TET, and ERT. On the other hand, 44 isolates (64.7%) were resistant to GEN, two (2.9%) to NAL, and only one (1.4%) isolate was resistant to ENO. Only three resistance patterns were observed among the isolates: GEN (42/68), NAL‐GEN (1/68), and NAL‐GEN‐ENO (1/68). The remaining 25 isolates were pan‐susceptible.

### 3.3 Whole genome sequencing analysis

All strains from rare serotypes (*n *= 68; 100%) harbored at least two antimicrobial resistance determinants (Table 2). The antimicrobial resistance genes (ARGs) *qnrB19* and *fosA7*, encoding resistance to quinolones and fosfomycin, respectively, were frequently identified among the isolates. In addition, it is worth noting that 60 (88.2%) isolates harbored *mdsA* and *mdsB* determinants, encoding multidrug and metal efflux proteins. The intrinsic aminoglycoside‐resistant determinant *aac*(*6′*)*‐Iaa* was detected in all isolates except one. Considering that this gene alone is not typically associated with phenotypic resistance to GEN (Salipante, 2003), isolates harboring no further aminoglycoside‐resistant determinants were susceptible to GEN in the phenotypic test (Table 1).

Out of 68 isolates, 67 (98.5%) isolates displayed a mutation in the codon 57 (Threonine→Serine) of the quinolone resistance‐determining region *parC* region. No mutations in *gyrA*, *gyrB*, and *parE* were observed. Additionally, incompatibility group plasmids such as IncFII (S), IncFII (Cf) Col (pHAD28), and IncFII (SARC14) were identified. Two *S*. Carrau and two *S*. Urbana were found to harbor the IncX3 plasmid (Table 2), known to harbor clinically important ARGs such as *bla*_NDM‐1_ and *bla*_KPC‐2_.

### 3.4 Multi‐locus sequence typing

Genomic prediction revealed the occurrence of 22 international sequence types (STs) as summarized in Table 1. *Salmonella* Carrau (ST226), *S*. Oran (ST965), and *S*. Urbana (ST754) showed consistent profiles, particularly in the serovar‐ST ratio. To the best of our knowledge, only ST226 has been previously reported in Brazil, with all other STs being described for the first time in this study. The 22 novel STs detected in this study were queried against the EnteroBase *Salmonella* database to assess their prevalence and relatedness to existing global lineages. None of these STs had been previously reported in EnteroBase, indicating their potential uniqueness to this geographic region or under‐sampled environmental niches. Phylogenetic analyses revealed that several of these novel STs cluster closely with known international lineages, suggesting possible evolutionary links or common ancestors. However, the distinct allelic profiles emphasize the genetic diversity present in Brazilian environmental *Salmonella* populations and highlight the importance of ongoing surveillance and genome sequencing efforts to capture the full breadth of *Salmonella* diversity worldwide.

### 3.5 Phylogenetic analysis

**FIGURE 2:**
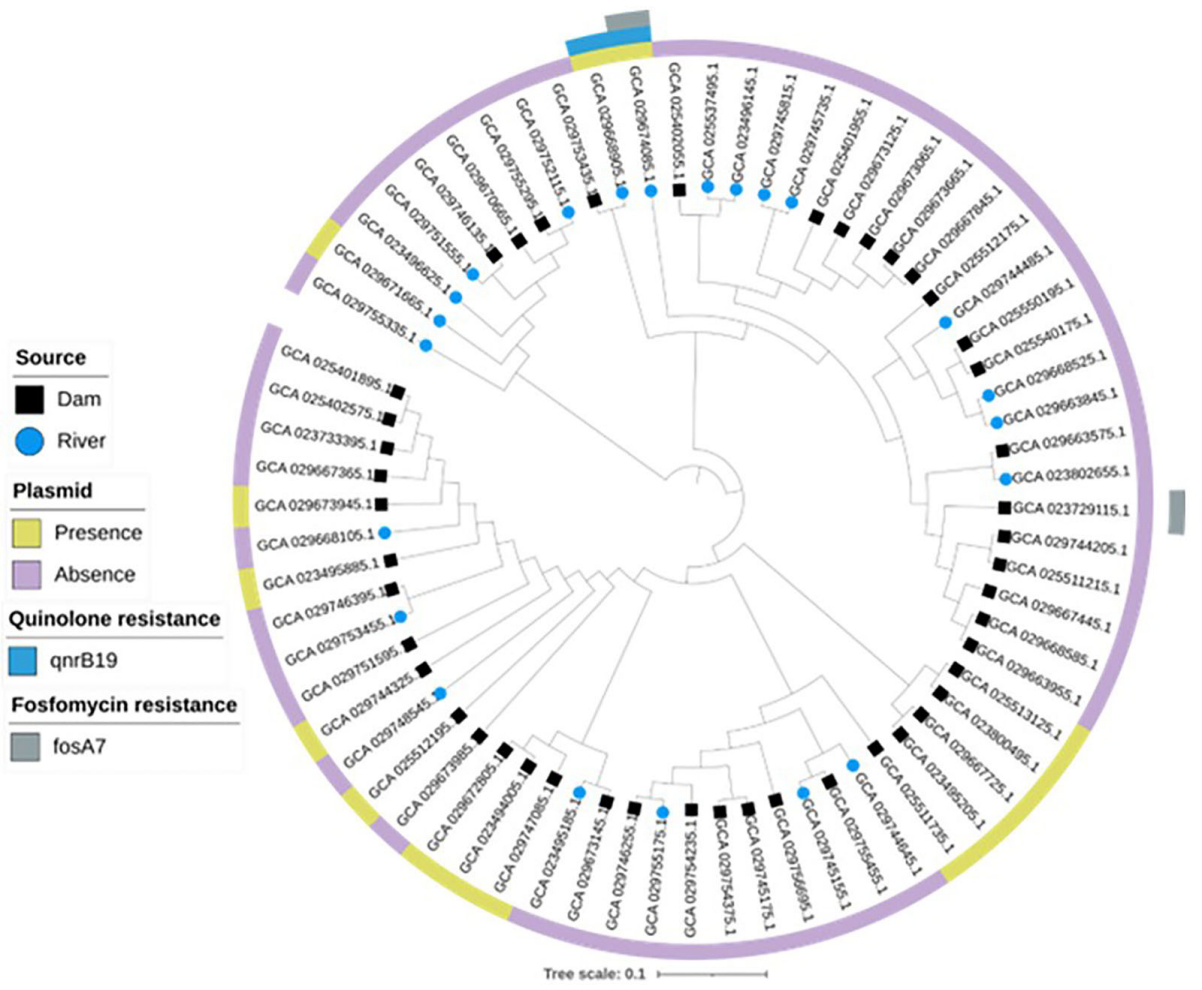
Maximum‐likelihood phylogenetic tree of 68 rare *Salmonella enterica* strains isolated from environmental water. The resulting phylogeny was rooted at midpoint.

Each serovar was represented by a monophyletic clade on the reconstructed phylogeny (Figure 2). We observed that the main drivers for cluster analysis were serovar and STs, since all isolates were clustered together by serovar and ST, and not by resistance profile, year of isolation, source, or geographic location. These findings are in agreement with previous studies (D. F. Monte et al., 2019; D. F. M. Monte et al., 2021). Isolates belonging to *S*. Carrau, *S*. Oran, *S*. Urbana, *S*. Gaminara, *S*. Othmarschen, *S*. Businga, *S*. Bullbay, *S*. Molade, *S*. Lomita, *S*. Mikawasima, *S*. Langenhorn, *S*. I 16:e,h:e,n,z15, *S*. II 43:z4,z23:‐ or IIIa 43:z4,z23:‐ or Farmingdale or IV 43:z4,z23:‐, *S*. I 18:d:‐, *S*. I 7:l,v:‐, *S*. I 7:k:‐, *S*. I 7:‐:1,5, *S*. I 16:r:e,n,z15, *S*. l,v:e,n,z15, and *S*. IV 45:g,z51:‐ were not clustered with international clones across the phylogeny, suggesting that these isolates are independent lineages circulating in Brazil. Conversely, *S*. Kiambu nested with strains from different countries (the United States, Canada, Mexico, China, the United Kingdom, Northern Ireland, and the Netherlands) and sources (clinical and non‐clinical) (PDG000000002.2708).

## 4 DISCUSSION

### 4.1 Isolation of rare *Salmonella enterica* serovars

Although the isolation of uncommon *S. enterica* serovars continues to be reported (D. F. M. Monte et al., 2021; Octavia et al., 2019; Tankson et al., 2006), there are relatively few studies regarding these serovars in Brazil and abroad. *Salmonella* Carrau was primarily identified in swine in 1944 (Hormaeche et al., 1944). Nearly six decades apart, this serovar was reported in Brazil from asymptomatic dogs (Maciel et al., 2004), human (Loureiro et al., 2010), turtle (Sousa et al., 2011), and swine plasma (D. F. M. Monte et al., 2021). Moreover, *S*. Carrau strains were isolated from ham, sausage, meat, and cheese in Colombia (Karczmarczyk et al., 2010). The diversity of sources from which *S*. Carrau has been isolated demonstrates its ability to adapt to new environments and potentially infect different hosts. Most reported isolates harbored the same *parC* substitution (Thr57→Ser) that was also detected in this study and belonged to the same ST226 previously reported in Brazil by Monte et al. (2021), indicating a common genetic lineage or clonal group circulating in Brazil.

*Salmonella* Urbana has been primarily associated with human clinical cases, mostly in children (Devi & Murray, 1991; Kocianová et al., 2010; Rimhanen‐Finne et al., 2010; Saba et al., 2013; Sirinavin et al., 1991; Williams et al., 2015). Additionally, this serovar has also been reported in turtle tank water (Kocianová et al., 2010), retail meat samples in Lagos, Nigeria (Smith et al., 2016), and raw cashews linked to a salmonellosis outbreak associated with a fermented cashew Brie analog in the United States (Louvau & Harris, 2023).

The presence of *S*. Gaminara is limited to a few reports, including an outbreak in individuals who consumed unpasteurized orange juice in the United States (Parish, 1998). Gopee et al. (Gopee et al., 2000) cultured resistant *S*. Gaminara strains from captive wildlife in Trinidad, while Durango et al. (2004) isolated 9% of *S*. Gaminara from fast food outlets in Colombia. Pantozzi et al. (Pantozzi et al., 2010) reported susceptible strains from domestic animals in Argentina, and most recently, Bonardi et al. (Bonardi et al., 2019) detected *S*. Gaminara from wild boars in Italy.

The first report of *S*. Lille was first isolated from chicken in 1954 (Kauffmann et al., 1954), and around two decades later in Iraq (Al‐Hindawi & Rished, 1979). More recent studies reported *S*. Lille strain in ground beef (Gupta et al., 2016b), lymph nodes of healthy cattle at slaughter (Webb et al., 2017), and backyard chickens in Argentina (Rodríguez et al., 2018).

To date, there is only one study reporting *S*. Freetown, which was recovered from a stool sample of a child in Argentina (Caffer et al., 1996). Similarly, there is only one report of *S*. Businga isolated in the Belgian Congo (Van Oye & Lucasse, 1957).

Although *S*. Molade has been rarely reported, this serovar has been recovered from different sources worldwide, such as pasturma beef sausages (Abbar & Tahir, 1989), eggshell (Suresh et al., 2006), and chicken and duck (N. Kumar et al., 2019).

Among the few studies reporting *S*. Kiambu, a case of transmission of *S*. Kiambu from feral pigeons to humans has been documented (Lacassin et al., 1995). The remaining studies indicate the presence of this serovar in foodstuff (Bouchrif et al., 2009), western grey kangaroos (Potter et al., 2011), cattle (Gupta et al., 2016a), wastewater (Mhongole et al., 2017), papayas (Hassan et al., 2019), human feces (Asakura et al., 2020), dairy heifer calves (Aleri et al., 2022), and poultry production (Quinn et al., 2023). Consistent with our findings, *S*. Kiambu isolates from wastewater belonged to ST309 (Aleri et al., 2022) and were resistant to multiple antimicrobials (Mhongole et al., 2017). There is much to be deciphered about this serovar, given lineages of these isolates seem to be circulating overseas.

The first case of *S*. Lomita appears to be related to a cause of thoracic empyema (Bartos & Hejzlar, 1959). The remaining reports point to the presence of this serovar in poultry (A. A. Kumar & Sawhney, 1970), spondylodiscitis (Chevalier et al., 1993), badgers (Wilson et al., 2003), retropharyngeal abscess in a child (Su et al., 2003), pigeon (Osman et al., 2013), squabs (Osman et al., 2014), and fecal sample of a human patient (R. Li et al., 2020). The recovery of antimicrobial‐resistant *S*. Lomita isolates from clinical samples highlights the pathogenicity of this serovar for both humans and animals.

*Salmonella* Mikawasima has been reported in human sources (Bartlett et al., 1978), freshwater aquarium snail (Bartlett et al., 1978), human outbreaks free (Freeman et al., 2016; Myšková & Karpíšková, 2014; Synnott et al., 1993), freshwater reservoirs (Polo et al., 1999), hospital (Navarro, 2001), patients with sporadic diarrhea (Kaneko et al., 2007), and tilapia (Budiati et al., 2016).

Although *S*. Othmarschen has not been commonly isolated, some reports highlight its occurrence in calves (Allsup et al., 1969), nosocomial outbreak (Morosini et al., 1996), humans (Erdem et al., 2005), a funeral outbreak (Kim et al., 2007), ostrich (Keokilwe et al., 2015), and an iliacus abscess (Jha et al., 2016).

Rarely isolated, *S*. Adelaide was first reported in 1943 from a fatal case of enteritis in Adelaide (Atkinson, 1943). Shortly after, Atkinson et al. (1952) reported its occurrence in humans, rats, and lizards. Further studies reported *S*. Adelaide in a child with diarrhea (Varghese et al., 1969), feral goats (McOrist & Miller, 1981), meat products (Mehrabian & Jaberi, 2006), dead keet caecum (Boko et al., 2013), and egg farms (McWhorter & Chousalkar, 2015).

*Salmonella* Oran ST965 is an exceptionally rare serovar, as it has not been deposited in the Enterobase database and has not been previously described in Brazil or any other location. Our results indicate that the ST965 is conserved within this serovar, and the presence of AMR genes encoding resistance to aminoglycosides and quinolones demonstrates its potential to adapt by acquiring resistance mechanisms. Similarly, there is no report of *S*. Bullbay ST9402, except further two genomes deposited in Enterobase and three deposited in NCBI. Although this is an extremely rare serovar, two of these genomes (SAMN08437214 and SAMN07731350) originated from human clinical cases, indicating its pathogenicity to humans.

While there are no reports of *S*. Langenhorn in current literature, the presence of this serovar is limited to a single genome deposited in Enterobase, which is associated with an isolate recovered from a human in Germany in 1960. Four genomes (SAMN17761171, SAMN17216100, SAMN17296603, and SAMN12310202) of *S*. Farmingdale serovar have been deposited in NCBI. It is noteworthy that all these genomes are associated with clinical cases, indicating a potential public health concern as *S*. Farmingdale serovar is pathogenic to humans. Lastly, there are no reports for the remaining detected serovars *S*. I 16:e,h:e,n,z15, *S*. I 18:d:‐, *S*. I 7:l,v:‐, *S*. I 4:b:‐, *S*. I 4:‐:1,5, *S*. I 7:k:‐, *S*. I 7:‐:1,5, *S*. I 16:r:e,n,z15, *S*. l,v:e,n,z15, *S*. IV [1],53:g,z51:‐, *S*. IV 50:z4,z23:‐, *S*. I 3,10:d:‐, *S*. Somone or IV 6,7:z4,z24:‐, and *S*. IV 45:g,z51:‐.

### 4.2 Antimicrobial resistance in rare *S. enterica* serovars

The significance of the serovar lies not only in its infrequent isolation, but also in the emergence of antimicrobial resistance, particularly to quinolones, which was frequently identified in this study. Besides vertical AMR transmission, the presence of plasmid‐mediated quinolone resistance gene (*qnrB19*) highlights a potential concern in public health since this plasmid can be horizontally disseminated. The occurrence of rare serovars carrying important AMR genes speaks to the pathogenic potential of these uncommon *S. enterica* serovars. Our findings suggest that the occurrence of antimicrobial‐resistant *Salmonella* serovars in environmental water should be further monitored and addressed.

The detection of plasmid‐mediated resistance genes, including those associated with IncX3 plasmids, highlights the potential for horizontal gene transfer in aquatic environments. IncX3 plasmids are known vectors for the dissemination of clinically relevant antimicrobial resistance genes, facilitating their rapid spread among diverse bacterial populations (Guo et al., 2022). Aquatic environments may act as reservoirs and hotspots for the exchange of such plasmids, thereby enhancing the risk of AMR dissemination along the food chain and into human populations. This underscores the need for targeted surveillance of plasmid‐mediated resistance in environmental *Salmonella* and reinforces the importance of limiting the release of antibiotic residues and resistant bacteria into natural water systems.

### 4.3 Environmental factors

The presence of rare *S. enterica* serovars in rivers and reservoirs suggests several potential pathways by which these bacteria can enter agrifood systems. Contaminated surface water used for irrigation may introduce *S. enterica* to fresh produce, while water sources shared by livestock could facilitate cross‐contamination and colonization. Runoff from agricultural lands and livestock operations may also introduce these serovars into water bodies. To mitigate these risks, integrated water management practices, such as routine water quality monitoring, treatment of irrigation water, and the implementation of Good Agricultural Practices, are essential. Additionally, limiting livestock access to natural water bodies and controlling manure runoff can reduce environmental contamination.

The edaphoclimatic characteristics of the investigated semiarid region, including precipitation concentrated in specific periods, high temperatures, and pronounced seasonal water variability in reservoirs, may have significant implications for the ecology of *Salmonella* in aquatic environments. During the dry season, reduced water flow and increased evaporation can lead to higher concentrations of organic matter and competing microbiota. Previous studies conducted in different environments and matrices, such as soil, manure, and vegetables, have shown that these conditions can reduce the ability of *S. enterica* to remain viable in the environment (Franz et al., 2005; García et al., 2010). Conversely, heavy rainfall events, though infrequent, can cause surface runoff from agricultural and livestock areas, introducing fecal material and associated pathogens into nearby water bodies (Liu et al., 2018). Interestingly, a previous longitudinal study in the region identified rainfall and non‐ruminant farming in areas near the sampling sites to be positively associated with increased recovery of *S. enterica* (A. D. L. Rocha et al., 2025). Additionally, water scarcity may intensify the reuse of contaminated water for irrigation or animal husbandry, increasing the likelihood of *Salmonella* circulation between environmental, agricultural, and human domains. These semiarid conditions underscore the importance of targeted monitoring and water management strategies, particularly in regions where water use intersects with food production.

### 4.4 Implications for public health and water quality management

Results emphasize the need for continued surveillance, particularly in low‐resource settings. Current surveillance frameworks in many such areas are either absent or insufficiently equipped to detect emerging or rare pathogens, especially those with public health significance. This study underscores the importance of integrating genomic surveillance into existing water quality programs. Policy initiatives should prioritize risk‐based monitoring approaches that consider proximity to livestock, waste discharge, and irrigation zones. In addition, guidelines must be adapted to address not only traditional fecal indicators but also specific foodborne pathogens and mobile AMR genes. Investments in affordable, field‐deployable diagnostic tools and training of local personnel are critical to enabling early detection and timely interventions. Strengthening these capacities can reduce the risk of pathogen transmission through agrifood systems and help prevent waterborne outbreaks in vulnerable communities.

### 4.5 Limitations and suggestions for future research

Several limitations must be acknowledged in our study. Sample size (*n* = 230) and the small number of rare *S. enterica* isolates recovered (*n* = 68) limit generalization of our findings. Sampling sites close to agricultural and livestock activities might have introduced selection bias, compromising the assessment of natural surface waters as reservoirs of rare *S. enterica* serovars. Moreover, we sampled shoreline access points only; we did not include interior transects or depth‐stratified profiles, which could provide important information about *Salmonella* distribution in water bodies. Lastly, continuous, year‐round sampling was not feasible due to multi‐basin logistics, access permissions, and field/laboratory capacity. Accordingly, we interpreted results as risk‐based detections rather than basin‐level prevalence estimates and refrain from formal seasonal inference. As such, we could not robustly assess seasonality, contamination dynamics, or spatial gradients within each reservoir.

Future work could employ year‐round, probabilistic sampling that includes shoreline‐to‐interior and depth‐stratified profiles (shoreline vs. pelagic; surface vs. depth); integrate enumeration techniques (e.g., large‐volume most probable number or viability‐informed quantitative or digital droplet PCR approaches); measure environmental covariates (pH, temperature, turbidity, nutrients); and use long‐read sequencing to resolve plasmid context. Such designs are required to estimate prevalence and loads, evaluate seasonal and hydrological drivers, and inform quantitative risk management for irrigation and livestock water. Such knowledge is necessary for a continuous improvement of the quality and safety of waters used in agrifood systems.

Despite the limited number of isolates, these belonged to a broad range of different serovars (*n* = 30), even though there were no screening tests for serovar prediction before selection of isolates for sequencing, such as repetitive extragenic palindromic sequence‐based PCR (rep‐PCR) or slide agglutination using *Salmonella* grouping antisera. Therefore, these findings indicate that the occurrence of other rare *Salmonella* serovars in water samples is far from a comprehensive characterization.

## 5 CONCLUSIONS

This study provides risk‐based evidence that rare *S. enterica* serovars occur in surface‐water reservoirs within a semiarid, agriculturally active region of northeastern Brazil. Using enrichment‐based detection and whole‐genome sequencing, we documented 30 infrequent serovars among 68 isolates and described their antimicrobial resistance determinants (e.g., frequent *parC* p.T57S and occasional *qnrB19* and *fosA7*) and plasmid replicons (including IncX3 in a subset of isolates). Detection of a diversity of rare *S. enterica* ssp. *enterica* strains from rivers and reservoirs highlights the importance of understanding the distribution of these potentially pathogenic bacteria in water sources. It emphasizes the need for continued surveillance and risk assessment, particularly in low‐resource settings. It also emphasizes the need for effective water quality monitoring policies and water management strategies to safeguard public health and prevent the spread of antimicrobial resistance. This study contributes to our knowledge of the epidemiology of *S. enterica* in environmental settings.

## AUTHOR CONTRIBUTIONS

**Alan Douglas de Lima Rocha**: Data curation; formal analysis; investigation; writing—original draft. **Daniel F. M. Monte**: Conceptualization; data curation; formal analysis; investigation; methodology; visualization; writing—original draft. **Laiorayne Araújo de Lima**: Investigation; writing—original draft. **Nádyra Jerônimo da Silva**: Investigation; writing—original draft. **Walter Esfrain Pereira**: Formal analysis; validation. **Patrícia Emília Naves Givisiez**: Validation; writing—review and editing. **Xinyang Huang**: Validation; writing—review and editing. **Zhao Chen**: Validation; writing—review and editing. **Eric W. Brown**: Validation; writing—review and editing. **Marc W. Allard**: Validation; writing—review and editing. **Rebecca L. Bell**: Validation; writing—review and editing. **Magaly Toro**: Supervision; validation; writing—review and editing. **Jianghong Meng**: Funding acquisition; project administration; supervision; writing—review and editing. **Celso José Bruno de Oliveira**: Funding acquisition; project administration; resources; supervision; writing—review and editing.

## CONFLICT OF INTEREST STATEMENT

The authors declare no conflicts of interest.

## Supporting information




**Table S1**. Sampling dates and geographical coordinates for all sampling events.

## Data Availability

The datasets analyzed during the current study are available in the NCBI repository (https://www.ncbi.nlm.nih.gov/sra) under the Bioproject PRJNA560080 (Joint Institute For Food Safety And Applied Nutrition: Whole Genome Sequencing of *Salmonella enterica* isolated from water samples). Genome access numbers are provided in Table 1 (available genomes that have been included in some genomic analyses). Further metadata will be made available on request.
