# Supplementary material for: Surface water as a source of rare Salmonella enterica serovars in semiarid northeastern Brazil
Source: J Environ Qual. 2025 Oct 21;54(6):1996–2013. doi: 10.1002/jeq2.70098 (PMC12593296; doi:10.1002/jeq2.70098)
Supplement: Supplementary file 1 — Table S1. Sampling dates and geographical coordinates for all sampling events. [file JEQ2-54-1996-s001.docx]

|  | | | |  |  |
| --- | --- | --- | --- | --- | --- |
| **Table S1. Sampling dates and geographical coordinates for all sampling events** | | | | | |
| **PROJECT YEAR** | **WATERSHED** | **CITY** | **COORDINATE** | **WATER SOURCE** | **DATE** |
| 2020/2021 | MAMANGUAPE | AREIA | 6°55'45.2"S 35°40'43.6"W | RESERVOIR | 2021-01-21 |
| 2020/2021 | MAMANGUAPE | AREIA | 6°55'07.6"S 35°40'41.7"W | RESERVOIR | 2021-01-21 |
| 2020/2021 | MAMANGUAPE | AREIA | 6°55'03.9"S 35°40'32.2"W | RESERVOIR | 2021-01-21 |
| 2020/2021 | MAMANGUAPE | ARAÇAGÍ | 6°51'16.4"S 35°18'03.9"W | RESERVOIR | 2021-01-22 |
| 2020/2021 | MAMANGUAPE | ARAÇAGÍ | 6°51'47.1"S 35°21'32.1"W | RIVER | 2021-01-22 |
| 2020/2021 | MAMANGUAPE | ARAÇAGÍ | 6°51'27.8"S 35°22'02.5"W | RIVER | 2021-01-22 |
| 2020/2021 | CURIMATAU | BANANEIRAS | 6°39'35.5"S 35°40'34.0"W | RESERVOIR | 2021-01-25 |
| 2020/2021 | CURIMATAU | BANANEIRAS | 6°39'30.3"S 35°40'39.4"W | RESERVOIR | 2021-01-25 |
| 2020/2021 | PARAÍBA | BOQUEIRÃO | 7°31'15.9"S 36°09'57.7"W | RESERVOIR | 2021-01-27 |
| 2020/2021 | PARAÍBA | BOQUEIRÃO | 7°30'39.0"S 36°09'25.4"W | RESERVOIR | 2021-01-27 |
| 2020/2021 | PARAÍBA | BOQUEIRÃO | 7°30'13.0"S 36°08'42.7"W | RESERVOIR | 2021-01-27 |
| 2020/2021 | PARAÍBA | ITATUBA | 7°25'56.5"S 35°33'41.7"W | RESERVOIR | 2021-01-28 |
| 2020/2021 | PARAÍBA | ITATUBA | 7°25'57.0"S 35°33'39.2"W | RESERVOIR | 2021-01-28 |
| 2020/2021 | PARAÍBA | ITATUBA | 7°26'38.2"S 35°33'36.7"W | RIVER | 2021-01-28 |
| 2020/2021 | PIANCÓ | COREMAS | 7°01'26.4"S 37°57'11.1"W | RESERVOIR | 2021-02-16 |
| 2020/2021 | PIANCÓ | COREMAS | 7°01'34.9"S 37°56'36.7"W | RESERVOIR | 2021-02-16 |
| 2020/2021 | PIANCÓ | COREMAS | 7°04'34.2"S 37°58'43.3"W | RESERVOIR | 2021-02-16 |
| 2020/2021 | PIANCÓ | COREMAS | 7°01'00.3"S 37°59'07.8"W | RIVER | 2021-02-16 |
| 2020/2021 | PIRANHAS | SOUSA | 6°50'39.7"S 38°18'56.1"W | RESERVOIR | 2021-02-17 |
| 2020/2021 | PIRANHAS | SOUSA | 6°50'45.1"S 38°18'40.1"W | RESERVOIR | 2021-02-17 |
| 2020/2021 | PIRANHAS | SOUSA | 6°51'22.5"S 38°21'05.5"W | RESERVOIR | 2021-02-17 |
| 2020/2021 | PIRANHAS | CONDADO | 6°54'28.3"S 37°35'07.3"W | RESERVOIR | 2021-02-18 |
| 2020/2021 | PIRANHAS | CONDADO | 6°55'17.8"S 37°35'25.2"W | RESERVOIR | 2021-02-18 |
| 2020/2021 | PIRANHAS | CONDADO | 6°54'53.3"S 37°35'04.1"W | RESERVOIR | 2021-02-18 |
| 2020/2021 | MAMANGUAPE | AREIA | 6°55'45.2"S 35°40'43.6"W | RESERVOIR | 2021-03-01 |
| 2020/2021 | MAMANGUAPE | AREIA | 6°55'07.6"S 35°40'41.7"W | RESERVOIR | 2021-03-01 |
| 2020/2021 | MAMANGUAPE | AREIA | 6°55'03.9"S 35°40'32.2"W | RESERVOIR | 2021-03-01 |
| 2020/2021 | CURIMATAU | BANANEIRAS | 6°39'35.5"S 35°40'34.0"W | RESERVOIR | 2021-03-03 |
| 2020/2021 | CURIMATAU | BANANEIRAS | 6°39'30.3"S 35°40'39.4"W | RESERVOIR | 2021-03-03 |
| 2020/2021 | MAMANGUAPE | ARAÇAGÍ | 6°51'16.4"S 35°18'03.9"W | RESERVOIR | 2021-03-05 |
| 2020/2021 | MAMANGUAPE | ARAÇAGÍ | 6°51'47.1"S 35°21'32.1"W | RIVER | 2021-03-05 |
| 2020/2021 | MAMANGUAPE | ARAÇAGÍ | 6°51'27.8"S 35°22'02.5"W | RIVER | 2021-03-05 |
| 2020/2021 | PARAÍBA | ITATUBA | 7°25'56.5"S 35°33'41.7"W | RESERVOIR | 2021-03-09 |
| 2020/2021 | PARAÍBA | ITATUBA | 7°25'57.0"S 35°33'39.2"W | RESERVOIR | 2021-03-09 |
| 2020/2021 | PARAÍBA | ITATUBA | 7°26'38.2"S 35°33'36.7"W | RIVER | 2021-03-09 |
| 2020/2021 | PARAÍBA | BOQUEIRÃO | 7°31'15.9"S 36°09'57.7"W | RESERVOIR | 2021-03-11 |
| 2020/2021 | PARAÍBA | BOQUEIRÃO | 7°30'39.0"S 36°09'25.4"W | RESERVOIR | 2021-03-11 |
| 2020/2021 | PARAÍBA | BOQUEIRÃO | 7°30'13.0"S 36°08'42.7"W | RESERVOIR | 2021-03-11 |
| 2020/2021 | PIRANHAS | CONDADO | 6°54'28.3"S 37°35'07.3"W | RESERVOIR | 2021-03-16 |
| 2020/2021 | PIRANHAS | CONDADO | 6°55'17.8"S 37°35'25.2"W | RESERVOIR | 2021-03-16 |
| 2020/2021 | PIRANHAS | CONDADO | 6°54'53.3"S 37°35'04.1"W | RESERVOIR | 2021-03-16 |
| 2020/2021 | PIRANHAS | CAJAZEIRAS | 6°59'13.3"S 38°27'19.0"W | RESERVOIR | 2021-03-17 |
| 2020/2021 | PIRANHAS | CAJAZEIRAS | 6°59'29.6"S 38°27'23.0"W | RESERVOIR | 2021-03-17 |
| 2020/2021 | PIRANHAS | CAJAZEIRAS | 6°58'57.6"S 38°27'12.1"W | RIVER | 2021-03-17 |
| 2020/2021 | PIANCÓ | COREMAS | 7°01'27.4"S 37°57'11.8"W | RESERVOIR | 2021-03-18 |
| 2020/2021 | PIANCÓ | COREMAS | 7°01'36.3"S 37°56'34.4"W | RESERVOIR | 2021-03-18 |
| 2020/2021 | PIANCÓ | COREMAS | 7°01'25.7"S 37°56'49.5"W | RESERVOIR | 2021-03-18 |
| 2020/2021 | PIANCÓ | COREMAS | 7°04'34.2"S 37°58'43.3"W | RESERVOIR | 2021-03-18 |
| 2020/2021 | PIANCÓ | COREMAS | 7°01'00.3"S 37°59'07.8"W | RIVER | 2021-03-18 |
| 2020/2021 | PIRANHAS | SOUSA | 6°50'39.7"S 38°18'56.1"W | RESERVOIR | 2021-03-19 |
| 2020/2021 | PIRANHAS | SOUSA | 6°50'45.1"S 38°18'40.1"W | RESERVOIR | 2021-03-19 |
| 2020/2021 | PIRANHAS | SOUSA | 6°51'22.5"S 38°21'05.5"W | RESERVOIR | 2021-03-19 |
| 2020/2021 | MAMANGUAPE | AREIA | 6°55'45.2"S 35°40'43.6"W | RESERVOIR | 2021-03-29 |
| 2020/2021 | MAMANGUAPE | AREIA | 6°55'07.6"S 35°40'41.7"W | RESERVOIR | 2021-03-29 |
| 2020/2021 | MAMANGUAPE | AREIA | 6°55'03.9"S 35°40'32.2"W | RESERVOIR | 2021-03-29 |
| 2020/2021 | MAMANGUAPE | ARAÇAGÍ | 6°51'16.4"S 35°18'03.9"W | RESERVOIR | 2021-03-31 |
| 2020/2021 | MAMANGUAPE | ARAÇAGÍ | 6°51'47.1"S 35°21'32.1"W | RIVER | 2021-03-31 |
| 2020/2021 | MAMANGUAPE | ARAÇAGÍ | 6°51'27.8"S 35°22'02.5"W | RIVER | 2021-03-31 |
| 2020/2021 | CURIMATAU | BANANEIRAS | 6°39'33.8"S 35°40'34.9"W | RESERVOIR | 2021-04-05 |
| 2020/2021 | CURIMATAU | BANANEIRAS | 6°39'30.6"S 35°40'39.7"W | RESERVOIR | 2021-04-05 |
| 2020/2021 | CURIMATAU | BANANEIRAS | 6°39'42.1"S 35°40'35.4"W | RESERVOIR | 2021-04-05 |
| 2020/2021 | PARAÍBA | ITATUBA | 7°25'56.5"S 35°33'41.7"W | RESERVOIR | 2021-04-07 |
| 2020/2021 | PARAÍBA | ITATUBA | 7°25'57.0"S 35°33'39.2"W | RESERVOIR | 2021-04-07 |
| 2020/2021 | PARAÍBA | ITATUBA | 7°26'38.2"S 35°33'36.7"W | RIVER | 2021-04-07 |
| 2020/2021 | PARAÍBA | BOQUEIRÃO | 7°31'15.9"S 36°09'57.7"W | RESERVOIR | 2021-04-09 |
| 2020/2021 | PARAÍBA | BOQUEIRÃO | 7°30'39.0"S 36°09'25.4"W | RESERVOIR | 2021-04-09 |
| 2020/2021 | PARAÍBA | BOQUEIRÃO | 7°30'13.0"S 36°08'42.7"W | RESERVOIR | 2021-04-09 |
| 2020/2021 | PIRANHAS | CONDADO | 6°54'28.3"S 37°35'07.3"W | RESERVOIR | 2021-04-13 |
| 2020/2021 | PIRANHAS | CONDADO | 6°55'17.8"S 37°35'25.2"W | RESERVOIR | 2021-04-13 |
| 2020/2021 | PIRANHAS | CONDADO | 6°54'53.3"S 37°35'04.1"W | RESERVOIR | 2021-04-13 |
| 2020/2021 | PIRANHAS | CAJAZEIRAS | 6°59'13.3"S 38°27'19.0"W | RESERVOIR | 2021-04-14 |
| 2020/2021 | PIRANHAS | CAJAZEIRAS | 6°59'29.6"S 38°27'23.0"W | RESERVOIR | 2021-04-14 |
| 2020/2021 | PIRANHAS | CAJAZEIRAS | 6°58'57.6"S 38°27'12.1"W | RIVER | 2021-04-14 |
| 2020/2021 | PIANCÓ | COREMAS | 7°01'27.4"S 37°57'11.8"W | RESERVOIR | 2021-04-15 |
| 2020/2021 | PIANCÓ | COREMAS | 7°01'36.3"S 37°56'34.4"W | RESERVOIR | 2021-04-15 |
| 2020/2021 | PIANCÓ | COREMAS | 7°01'25.7"S 37°56'49.5"W | RESERVOIR | 2021-04-15 |
| 2020/2021 | PIANCÓ | COREMAS | 7°04'34.2"S 37°58'43.3"W | RESERVOIR | 2021-04-15 |
| 2020/2021 | PIANCÓ | COREMAS | 7°01'00.3"S 37°59'07.8"W | RIVER | 2021-04-15 |
| 2020/2021 | PIRANHAS | SOUSA | 6°50'39.7"S 38°18'56.1"W | RESERVOIR | 2021-04-16 |
| 2020/2021 | PIRANHAS | SOUSA | 6°50'45.1"S 38°18'40.1"W | RESERVOIR | 2021-04-16 |
| 2020/2021 | PIRANHAS | SOUSA | 6°51'22.5"S 38°21'05.5"W | RESERVOIR | 2021-04-16 |
| 2020/2021 | PARAÍBA | BOQUEIRÃO | 7°31'15.9"S 36°09'57.7"W | RESERVOIR | 2021-05-05 |
| 2020/2021 | PARAÍBA | BOQUEIRÃO | 7°30'39.0"S 36°09'25.4"W | RESERVOIR | 2021-05-05 |
| 2020/2021 | PARAÍBA | BOQUEIRÃO | 7°30'13.0"S 36°08'42.7"W | RESERVOIR | 2021-05-05 |
| 2020/2021 | MAMANGUAPE | AREIA | 6°55'45.2"S 35°40'43.6"W | RESERVOIR | 2021-05-06 |
| 2020/2021 | MAMANGUAPE | AREIA | 6°55'07.6"S 35°40'41.7"W | RESERVOIR | 2021-05-06 |
| 2020/2021 | MAMANGUAPE | AREIA | 6°55'03.9"S 35°40'32.2"W | RESERVOIR | 2021-05-06 |
| 2020/2021 | CURIMATAU | BANANEIRAS | 6°39'33.8"S 35°40'34.9"W | RESERVOIR | 2021-05-06 |
| 2020/2021 | CURIMATAU | BANANEIRAS | 6°39'30.6"S 35°40'39.7"W | RESERVOIR | 2021-05-06 |
| 2020/2021 | MAMANGUAPE | ARAÇAGÍ | 6°51'16.4"S 35°18'03.9"W | RESERVOIR | 2021-05-07 |
| 2020/2021 | MAMANGUAPE | ARAÇAGÍ | 6°51'47.1"S 35°21'32.1"W | RIVER | 2021-05-07 |
| 2020/2021 | MAMANGUAPE | ARAÇAGÍ | 6°51'27.8"S 35°22'02.5"W | RIVER | 2021-05-07 |
| 2020/2021 | PARAÍBA | ITATUBA | 7°25'56.5"S 35°33'41.7"W | RESERVOIR | 2021-05-08 |
| 2020/2021 | PARAÍBA | ITATUBA | 7°25'57.0"S 35°33'39.2"W | RESERVOIR | 2021-05-08 |
| 2020/2021 | PARAÍBA | ITATUBA | 7°26'38.2"S 35°33'36.7"W | RIVER | 2021-05-08 |
| 2020/2021 | PIRANHAS | CONDADO | 6°54'28.3"S 37°35'07.3"W | RESERVOIR | 2021-05-11 |
| 2020/2021 | PIRANHAS | CONDADO | 6°55'17.8"S 37°35'25.2"W | RESERVOIR | 2021-05-11 |
| 2020/2021 | PIRANHAS | CONDADO | 6°54'53.3"S 37°35'04.1"W | RESERVOIR | 2021-05-11 |
| 2020/2021 | PIRANHAS | CAJAZEIRAS | 6°59'13.3"S 38°27'19.0"W | RESERVOIR | 2021-05-12 |
| 2020/2021 | PIRANHAS | CAJAZEIRAS | 6°59'29.6"S 38°27'23.0"W | RESERVOIR | 2021-05-12 |
| 2020/2021 | PIRANHAS | CAJAZEIRAS | 6°58'57.6"S 38°27'12.1"W | RIVER | 2021-05-12 |
| 2020/2021 | PIANCÓ | COREMAS | 7°01'27.4"S 37°57'11.8"W | RESERVOIR | 2021-05-13 |
| 2020/2021 | PIANCÓ | COREMAS | 7°01'36.3"S 37°56'34.4"W | RESERVOIR | 2021-05-13 |
| 2020/2021 | PIANCÓ | COREMAS | 7°01'25.7"S 37°56'49.5"W | RESERVOIR | 2021-05-13 |
| 2020/2021 | PIANCÓ | COREMAS | 7°04'34.2"S 37°58'43.3"W | RESERVOIR | 2021-05-13 |
| 2020/2021 | PIANCÓ | COREMAS | 7°01'00.3"S 37°59'07.8"W | RIVER | 2021-05-13 |
| 2020/2021 | PIRANHAS | SOUSA | 6°50'39.7"S 38°18'56.1"W | RESERVOIR | 2021-05-14 |
| 2020/2021 | PIRANHAS | SOUSA | 6°50'45.1"S 38°18'40.1"W | RESERVOIR | 2021-05-14 |
| 2020/2021 | PIRANHAS | SOUSA | 6°51'22.5"S 38°21'05.5"W | RESERVOIR | 2021-05-14 |
| 2020/2021 | MAMANGUAPE | AREIA | 6°55'45.2"S 35°40'43.6"W | RESERVOIR | 2021-05-25 |
| 2020/2021 | MAMANGUAPE | AREIA | 6°55'07.6"S 35°40'41.7"W | RESERVOIR | 2021-05-25 |
| 2020/2021 | MAMANGUAPE | AREIA | 6°55'03.9"S 35°40'32.2"W | RESERVOIR | 2021-05-25 |
| 2020/2021 | CURIMATAU | BANANEIRAS | 6°39'33.8"S 35°40'34.9"W | RESERVOIR | 2021-05-26 |
| 2020/2021 | CURIMATAU | BANANEIRAS | 6°39'30.6"S 35°40'39.7"W | RESERVOIR | 2021-05-26 |
| 2020/2021 | CURIMATAU | BANANEIRAS | 6°39'42.1"S 35°40'35.4"W | RESERVOIR | 2021-05-26 |
| 2020/2021 | MAMANGUAPE | ARAÇAGÍ | 6°51'16.4"S 35°18'03.9"W | RESERVOIR | 2021-05-31 |
| 2020/2021 | MAMANGUAPE | ARAÇAGÍ | 6°51'47.1"S 35°21'32.1"W | RIVER | 2021-05-31 |
| 2020/2021 | MAMANGUAPE | ARAÇAGÍ | 6°51'27.8"S 35°22'02.5"W | RIVER | 2021-05-31 |
| 2020/2021 | PARAÍBA | BOQUEIRÃO | 7°31'15.9"S 36°09'57.7"W | RESERVOIR | 2021-06-04 |
| 2020/2021 | PARAÍBA | BOQUEIRÃO | 7°30'39.0"S 36°09'25.4"W | RESERVOIR | 2021-06-04 |
| 2020/2021 | PARAÍBA | BOQUEIRÃO | 7°30'13.0"S 36°08'42.7"W | RESERVOIR | 2021-06-04 |
| 2020/2021 | PARAÍBA | ITATUBA | 7°25'56.5"S 35°33'41.7"W | RESERVOIR | 2021-06-07 |
| 2020/2021 | PARAÍBA | ITATUBA | 7°25'57.0"S 35°33'39.2"W | RESERVOIR | 2021-06-07 |
| 2020/2021 | PARAÍBA | ITATUBA | 7°26'38.2"S 35°33'36.7"W | RIVER | 2021-06-07 |
| 2020/2021 | PIRANHAS | CONDADO | 6°54'28.3"S 37°35'07.3"W | RESERVOIR | 2021-06-10 |
| 2020/2021 | PIRANHAS | CONDADO | 6°55'17.8"S 37°35'25.2"W | RESERVOIR | 2021-06-10 |
| 2020/2021 | PIRANHAS | CONDADO | 6°54'53.3"S 37°35'04.1"W | RESERVOIR | 2021-06-10 |
| 2020/2021 | PIRANHAS | SOUSA | 6°50'39.7"S 38°18'56.1"W | RESERVOIR | 2021-06-11 |
| 2020/2021 | PIRANHAS | SOUSA | 6°50'45.1"S 38°18'40.1"W | RESERVOIR | 2021-06-11 |
| 2020/2021 | PIRANHAS | SOUSA | 6°51'22.5"S 38°21'05.5"W | RESERVOIR | 2021-06-11 |
| 2020/2021 | PIRANHAS | CAJAZEIRAS | 6°59'13.3"S 38°27'19.0"W | RESERVOIR | 2021-06-12 |
| 2020/2021 | PIRANHAS | CAJAZEIRAS | 6°59'29.6"S 38°27'23.0"W | RESERVOIR | 2021-06-12 |
| 2020/2021 | PIRANHAS | CAJAZEIRAS | 6°58'57.6"S 38°27'12.1"W | RIVER | 2021-06-12 |
| 2020/2021 | PIANCÓ | COREMAS | 7°01'27.4"S 37°57'11.8"W | RESERVOIR | 2021-06-13 |
| 2020/2021 | PIANCÓ | COREMAS | 7°01'36.3"S 37°56'34.4"W | RESERVOIR | 2021-06-13 |
| 2020/2021 | PIANCÓ | COREMAS | 7°01'25.7"S 37°56'49.5"W | RESERVOIR | 2021-06-13 |
| 2020/2021 | PIANCÓ | COREMAS | 7°04'34.2"S 37°58'43.3"W | RESERVOIR | 2021-06-13 |
| 2020/2021 | PIANCÓ | COREMAS | 7°01'00.3"S 37°59'07.8"W | RIVER | 2021-06-13 |
| 2020/2021 | PARAÍBA | ITATUBA | 7°25'56.5"S 35°33'41.7"W | RESERVOIR | 2021-07-19 |
| 2020/2021 | PARAÍBA | ITATUBA | 7°25'57.0"S 35°33'39.2"W | RESERVOIR | 2021-07-19 |
| 2020/2021 | PARAÍBA | ITATUBA | 7°26'38.2"S 35°33'36.7"W | RIVER | 2021-07-19 |
| 2020/2021 | MAMANGUAPE | ARAÇAGÍ | 6°51'16.4"S 35°18'03.9"W | RESERVOIR | 2021-07-22 |
| 2020/2021 | MAMANGUAPE | ARAÇAGÍ | 6°51'47.1"S 35°21'32.1"W | RIVER | 2021-07-22 |
| 2020/2021 | MAMANGUAPE | ARAÇAGÍ | 6°51'27.8"S 35°22'02.5"W | RIVER | 2021-07-22 |
| 2020/2021 | PIANCÓ | COREMAS | 7°01'27.4"S 37°57'11.8"W | RESERVOIR | 2021-07-24 |
| 2020/2021 | PIANCÓ | COREMAS | 7°01'36.3"S 37°56'34.4"W | RESERVOIR | 2021-07-24 |
| 2020/2021 | PIANCÓ | COREMAS | 7°01'25.7"S 37°56'49.5"W | RESERVOIR | 2021-07-24 |
| 2020/2021 | PIRANHAS | SOUSA | 6°50'39.7"S 38°18'56.1"W | RESERVOIR | 2021-07-25 |
| 2020/2021 | PIRANHAS | SOUSA | 6°50'45.1"S 38°18'40.1"W | RESERVOIR | 2021-07-25 |
| 2020/2021 | PIRANHAS | SOUSA | 6°51'22.5"S 38°21'05.5"W | RESERVOIR | 2021-07-25 |
| 2020/2021 | PIRANHAS | CAJAZEIRAS | 6°59'13.3"S 38°27'19.0"W | RESERVOIR | 2021-07-25 |
| 2020/2021 | PIRANHAS | CAJAZEIRAS | 6°59'29.6"S 38°27'23.0"W | RESERVOIR | 2021-07-25 |
| 2020/2021 | PIRANHAS | CAJAZEIRAS | 6°58'57.6"S 38°27'12.1"W | RIVER | 2021-07-25 |
| 2020/2021 | MAMANGUAPE | ARAÇAGÍ | 6°51'16.4"S 35°18'03.9"W | RESERVOIR | 2021-08-25 |
| 2020/2021 | MAMANGUAPE | ARAÇAGÍ | 6°51'47.1"S 35°21'32.1"W | RIVER | 2021-08-25 |
| 2020/2021 | MAMANGUAPE | ARAÇAGÍ | 6°51'27.8"S 35°22'02.5"W | RIVER | 2021-08-25 |
| 2020/2021 | PARAÍBA | ITATUBA | 7°25'56.5"S 35°33'41.7"W | RESERVOIR | 2021-08-26 |
| 2020/2021 | PARAÍBA | ITATUBA | 7°25'57.0"S 35°33'39.2"W | RESERVOIR | 2021-08-26 |
| 2020/2021 | PARAÍBA | ITATUBA | 7°26'38.2"S 35°33'36.7"W | RIVER | 2021-08-26 |
| 2020/2021 | PIRANHAS | CAJAZEIRAS | 6°59'13.3"S 38°27'19.0"W | RESERVOIR | 2021-08-26 |
| 2020/2021 | PIRANHAS | CAJAZEIRAS | 6°59'29.6"S 38°27'23.0"W | RESERVOIR | 2021-08-26 |
| 2020/2021 | PIRANHAS | CAJAZEIRAS | 6°58'57.6"S 38°27'12.1"W | RIVER | 2021-08-26 |
| 2020/2021 | PIRANHAS | SOUSA | 6°50'39.7"S 38°18'56.1"W | RESERVOIR | 2021-08-28 |
| 2020/2021 | PIRANHAS | SOUSA | 6°50'45.1"S 38°18'40.1"W | RESERVOIR | 2021-08-28 |
| 2020/2021 | PIRANHAS | SOUSA | 6°51'22.5"S 38°21'05.5"W | RESERVOIR | 2021-08-28 |
| 2020/2021 | PIANCÓ | COREMAS | 7°01'27.4"S 37°57'11.8"W | RESERVOIR | 2021-08-28 |
| 2020/2021 | PIANCÓ | COREMAS | 7°01'36.3"S 37°56'34.4"W | RESERVOIR | 2021-08-28 |
| 2021/2022 | MAMANGUAPE | ARAÇAGÍ | 6°51'16.4"S 35°18'03.9"W | RESERVOIR | 2022-01-26 |
| 2021/2022 | MAMANGUAPE | ARAÇAGÍ | 6°51'47.1"S 35°21'32.1"W | RIVER | 2022-01-26 |
| 2021/2022 | MAMANGUAPE | ARAÇAGÍ | 6°51'27.8"S 35°22'02.5"W | RIVER | 2022-01-26 |
| 2021/2022 | MAMANGUAPE | ARAÇAGÍ | 6°51'16.4"S 35°18'03.9"W | RESERVOIR | 2022-02-10 |
| 2021/2022 | MAMANGUAPE | ARAÇAGÍ | 6°51'47.1"S 35°21'32.1"W | RIVER | 2022-02-10 |
| 2021/2022 | MAMANGUAPE | ARAÇAGÍ | 6°51'27.8"S 35°22'02.5"W | RIVER | 2022-02-10 |
| 2021/2022 | PARAÍBA | ITATUBA | 7°25'56.5"S 35°33'41.7"W | RESERVOIR | 2022-02-11 |
| 2021/2022 | PARAÍBA | ITATUBA | 7°25'57.0"S 35°33'39.2"W | RESERVOIR | 2022-02-11 |
| 2021/2022 | PARAÍBA | ITATUBA | 7°26'38.2"S 35°33'36.7"W | RIVER | 2022-02-11 |
| 2021/2022 | PIRANHAS | SOUSA | 6°50'39.7"S 38°18'56.2"W | RESERVOIR | 2022-02-17 |
| 2021/2022 | PIRANHAS | SOUSA | 7°01'35.8"S 37°56'34.8"W | RESERVOIR | 2022-02-17 |
| 2021/2022 | PIRANHAS | SOUSA | 6°50'51.6"S 38°18'33.7"W | RESERVOIR | 2022-02-17 |
| 2021/2022 | PIANCÓ | COREMAS | 7°01'26.4"S 37°57'11.1"W | RESERVOIR | 2022-02-17 |
| 2021/2022 | PIANCÓ | COREMAS | 7°01'34.9"S 37°56'36.7"W | RESERVOIR | 2022-02-17 |
| 2021/2022 | PIANCÓ | COREMAS | 7°01'27.4"S 37°57'11.8"W | RESERVOIR | 2022-02-17 |
| 2021/2022 | PIRANHAS | CAJAZEIRAS | 6°59'13.3"S 38°27'19.0"W | RESERVOIR | 2022-02-17 |
| 2021/2022 | PIRANHAS | CAJAZEIRAS | 6°59'29.6"S 38°27'23.0"W | RESERVOIR | 2022-02-17 |
| 2021/2022 | PIRANHAS | CAJAZEIRAS | 6°58'57.6"S 38°27'12.1"W | RIVER | 2022-02-17 |
| 2021/2022 | MAMANGUAPE | ARAÇAGÍ | 6°51'16.4"S 35°18'03.9"W | RESERVOIR | 2022-03-08 |
| 2021/2022 | MAMANGUAPE | ARAÇAGÍ | 6°51'47.1"S 35°21'32.1"W | RIVER | 2022-03-08 |
| 2021/2022 | MAMANGUAPE | ARAÇAGÍ | 6°51'27.8"S 35°22'02.5"W | RIVER | 2022-03-08 |
| 2021/2022 | PARAÍBA | ITATUBA | 7°25'56.5"S 35°33'41.7"W | RESERVOIR | 2022-03-09 |
| 2021/2022 | PARAÍBA | ITATUBA | 7°25'57.0"S 35°33'39.2"W | RESERVOIR | 2022-03-09 |
| 2021/2022 | PARAÍBA | ITATUBA | 7°26'38.2"S 35°33'36.7"W | RIVER | 2022-03-09 |
| 2021/2022 | PIRANHAS | SOUSA | 6°50'39.7"S 38°18'56.1"W | RESERVOIR | 2022-03-20 |
| 2021/2022 | PIRANHAS | SOUSA | 6°50'45.1"S 38°18'40.1"W | RESERVOIR | 2022-03-20 |
| 2021/2022 | PIRANHAS | SOUSA | 6°51'22.5"S 38°21'05.5"W | RESERVOIR | 2022-03-20 |
| 2021/2022 | PIANCÓ | COREMAS | 7°01'26.4"S 37°57'11.1"W | RESERVOIR | 2022-03-20 |
| 2021/2022 | PIANCÓ | COREMAS | 7°01'34.9"S 37°56'36.7"W | RESERVOIR | 2022-03-20 |
| 2021/2022 | PIANCÓ | COREMAS | 7°01'27.4"S 37°57'11.8"W | RESERVOIR | 2022-03-20 |
| 2021/2022 | PIRANHAS | CAJAZEIRAS | 6°59'13.3"S 38°27'19.0"W | RESERVOIR | 2022-03-20 |
| 2021/2022 | PIRANHAS | CAJAZEIRAS | 6°59'29.6"S 38°27'23.0"W | RESERVOIR | 2022-03-20 |
| 2021/2022 | PIRANHAS | CAJAZEIRAS | 6°58'57.6"S 38°27'12.1"W | RIVER | 2022-03-20 |
| 2021/2022 | PARAÍBA | ITATUBA | 7°25'56.5"S 35°33'41.7"W | RESERVOIR | 2022-04-05 |
| 2021/2022 | PARAÍBA | ITATUBA | 7°25'57.0"S 35°33'39.2"W | RESERVOIR | 2022-04-05 |
| 2021/2022 | PARAÍBA | ITATUBA | 7°26'38.2"S 35°33'36.7"W | RIVER | 2022-04-05 |
| 2021/2022 | MAMANGUAPE | ARAÇAGÍ | 6°51'16.4"S 35°18'03.9"W | RESERVOIR | 2022-04-07 |
| 2021/2022 | MAMANGUAPE | ARAÇAGÍ | 6°51'47.1"S 35°21'32.1"W | RIVER | 2022-04-07 |
| 2021/2022 | MAMANGUAPE | ARAÇAGÍ | 6°51'27.8"S 35°22'02.5"W | RIVER | 2022-04-07 |
| 2021/2022 | PIRANHAS | SOUSA | 6°50'39.7"S 38°18'56.1"W | RESERVOIR | 2022-04-24 |
| 2021/2022 | PIRANHAS | SOUSA | 6°50'45.1"S 38°18'40.1"W | RESERVOIR | 2022-04-24 |
| 2021/2022 | PIRANHAS | SOUSA | 6°51'22.5"S 38°21'05.5"W | RESERVOIR | 2022-04-24 |
| 2021/2022 | PIANCÓ | COREMAS | 7°01'26.4"S 37°57'11.1"W | RESERVOIR | 2022-04-24 |
| 2021/2022 | PIANCÓ | COREMAS | 7°01'34.9"S 37°56'36.7"W | RESERVOIR | 2022-04-24 |
| 2021/2022 | PIANCÓ | COREMAS | 7°01'27.4"S 37°57'11.8"W | RESERVOIR | 2022-04-24 |
| 2021/2022 | PIRANHAS | CAJAZEIRAS | 6°59'13.3"S 38°27'19.0"W | RESERVOIR | 2022-04-24 |
| 2021/2022 | PIRANHAS | CAJAZEIRAS | 6°59'29.6"S 38°27'23.0"W | RESERVOIR | 2022-04-24 |
| 2021/2022 | PIRANHAS | CAJAZEIRAS | 6°58'57.6"S 38°27'12.1"W | RIVER | 2022-04-24 |
| 2021/2022 | MAMANGUAPE | ARAÇAGÍ | 6°51'16.4"S 35°18'03.9"W | RESERVOIR | 2022-05-03 |
| 2021/2022 | MAMANGUAPE | ARAÇAGÍ | 6°51'47.1"S 35°21'32.1"W | RIVER | 2022-05-03 |
| 2021/2022 | MAMANGUAPE | ARAÇAGÍ | 6°51'27.8"S 35°22'02.5"W | RIVER | 2022-05-03 |
| 2021/2022 | PARAÍBA | ITATUBA | 7°25'56.5"S 35°33'41.7"W | RESERVOIR | 2022-05-04 |
| 2021/2022 | PARAÍBA | ITATUBA | 7°25'57.0"S 35°33'39.2"W | RESERVOIR | 2022-05-04 |
| 2021/2022 | PARAÍBA | ITATUBA | 7°26'38.2"S 35°33'36.7"W | RIVER | 2022-05-04 |
| 2021/2022 | PIRANHAS | SOUSA | 6°50'39.7"S 38°18'56.1"W | RESERVOIR | 2022-05-29 |
| 2021/2022 | PIRANHAS | SOUSA | 6°50'45.1"S 38°18'40.1"W | RESERVOIR | 2022-05-29 |
| 2021/2022 | PIRANHAS | SOUSA | 6°51'22.5"S 38°21'05.5"W | RESERVOIR | 2022-05-29 |
| 2021/2022 | PIANCÓ | COREMAS | 7°01'26.4"S 37°57'11.1"W | RESERVOIR | 2022-05-29 |
| 2021/2022 | PIANCÓ | COREMAS | 7°01'34.9"S 37°56'36.7"W | RESERVOIR | 2022-05-29 |
| 2021/2022 | PIANCÓ | COREMAS | 7°01'27.4"S 37°57'11.8"W | RESERVOIR | 2022-05-29 |
| 2021/2022 | PIRANHAS | CAJAZEIRAS | 6°59'13.3"S 38°27'19.0"W | RESERVOIR | 2022-05-29 |
| 2021/2022 | PIRANHAS | CAJAZEIRAS | 6°59'29.6"S 38°27'23.0"W | RESERVOIR | 2022-05-29 |
| 2021/2022 | PIRANHAS | CAJAZEIRAS | 6°58'57.6"S 38°27'12.1"W | RIVER | 2022-05-29 |
